# HUWE1 loss promotes stemness and drug resistance in CRC with dysregulated β-catenin destruction complex

**DOI:** 10.1038/s41420-025-02731-2

**Published:** 2025-10-06

**Authors:** Chanhaeng Lee, Sang-Hee Park, Inn-Oc Han, Sungjoo Kim Yoon

**Affiliations:** 1 Department of Medical Sciences, The Catholic University of Korea, 222 Banpo-daero, Seocho-gu, Seoul, 06591 Republic of Korea; 2 Department of Medical Life Sciences, College of Medicine, The Catholic University of Korea, 222 Banpo-daero, Seocho-gu, Seoul, 06591 Republic of Korea; 3 BK21 Center for Precision Medicine & Smart Engineering, Inha University, 100 Inha-ro, Michuhol-gu, Incheon, 22212 Republic of Korea; 4 Department of Biomedical Science, Program in Biomedical Science and Engineering, Inha University, 100 Inha-ro, Michuhol-gu, Incheon, 22212 Republic of Korea; 5 Department of Physiology and Biophysics, College of Medicine, Inha University, 100 Inha-ro, Michuhol-gu, Incheon, 22212 Republic of Korea

**Keywords:** Cancer stem cells, Epithelial-mesenchymal transition, Energy metabolism

## Abstract

Cancer stem cells (CSCs) are a key driver of tumor initiation, progression, and drug resistance in colorectal cancer (CRC). The Wnt/β-catenin signaling pathway, which is hyperactivated in nearly all CRC cases, plays a crucial role in CSC-related processes such as proliferation, epithelial-mesenchymal transition (EMT), and metastasis. In this study, we demonstrate that HUWE1 plays a critical regulator of Wnt/β-catenin signaling, similar to the β-catenin destruction complex. Under conditions of β-catenin destruction complex inactivation, most HUWE1 directly interacts with and ubiquitinates β-catenin. Conversely, when the destruction complex is active, HUWE1 targets upstream proteins for ubiquitination, thereby regulating Wnt/β-catenin signaling. This highlights HUWE1 as a pivotal regulator of Wnt/β-catenin signaling, particularly in CRC cases characterized by frequent *APC* mutations. Our findings further show that HUWE1 loss in CRC cells stabilizes β-catenin, enhancing CSC traits and promoting EMT. Additionally, HUWE1 depletion leads to excessive mitochondrial biogenesis, which contributes to drug resistance by supplying significant ATP levels to ATP-binding cassette (ABC) transporters. In conclusion, this study uncovers a previously unrecognized role of HUWE1 in regulating Wnt/β-catenin signaling and its impact on CRC. These insights may aid in identifying colorectal CSCs and developing targeted therapeutic strategies.

## Introduction

Colorectal cancer (CRC) is the third most common cancer worldwide [1]. Despite the diversification and advancements in cancer treatments leading to improved clinical outcomes for CRC patients, the prognosis remains poor due to the high heterogeneity and complexity of CRC [2, 3]. Cancer stem cells (CSCs) are a subpopulation within tumors that possess the abilities of self-renewal and differentiation [4]. CSCs have been reported to be involved in the initiation, progression, metastasis, and drug resistance of CRC [5]. Therefore, many studies have been conducted to understand how stemness-related signaling pathways are regulated in CSCs [6].

The Wnt/β-catenin signaling contributes to the generation and maintenance of CSCs even in cancer cells that lack stemness [7, 8]. Activation of the Wnt/β-catenin signaling induces the stabilization of β-catenin and its translocation to the nucleus, promoting cell proliferation, differentiation, and migration [9]. Wnt/β-catenin signaling plays a crucial role in embryonic development and tissue homeostasis, but its loss of regulation often leads to a broad range of cancers [10]. Nearly all CRCs harbor inactivating mutations in *APC*, highlighting hyperactivation of Wnt/β-catenin signaling as a key oncogenic driver [11].

HECT, UBA, and WWE domain containing E3 ligase 1 (HUWE1) is a large E3 ubiquitin ligase (482 kDa) that regulates a wide range of substrates through the ubiquitin-proteasome system (UPS), influencing diverse biological processes [12]. HUWE1 has been reported to downregulate the upstream of Wnt/β-catenin signaling by affecting the multimerization of dishevelled (Dvl) and promoting the degradation of pancreatic progenitor cell differentiation and proliferation factor (PPDPF) [13, 14]. Moreover, HUWE1 has been shown to directly interact with and degrade β-catenin under conditions of hyperactive Wnt/β-catenin signaling [15]. However, the comprehensive mechanism through which HUWE1 modulates overall Wnt/β-catenin signaling remains to be fully elucidated.

Mitochondrial biogenesis is a crucial cellular process involving the generation of new mitochondria, contributing to the maintenance of cellular biosynthesis and bioenergetics requirements [16]. Wnt/β-catenin signaling not only enhances the stemness and aggressiveness of cancer cells but also triggers mitochondrial biogenesis [17, 18]. The elevated mitochondrial biogenesis provides a substantial amount of ATP to the ATP-binding cassette (ABC) transporters, which are particularly overexpressed in CSCs, consequently leading to drug resistance [19, 20]. In our previous study, we observed that HUWE1-deficient CRC cells exhibited enhanced mitochondrial function, oxaliplatin resistance, and characteristics resembling CSCs [21]. However, these observations were made in a mixed cell population with various mutations in addition to HUWE1 deficiency. As a result, it remains unclear whether the loss of HUWE1 directly induces stemness and drug resistance in CRC.

In this study, we found that CRC cells with specific suppression of HUWE1 exhibit characteristics of CSCs and promote the epithelial-mesenchymal transition (EMT). We also discovered that HUWE1 regulates Wnt/β-catenin signaling similarly to the β-catenin destruction complex. Under conditions of β-catenin destruction complex inactivation, HUWE1 directly interacts with and ubiquitinates β-catenin. Conversely, when the β-catenin destruction complex is activated, HUWE1 interacts with upstream proteins such as Dvl and PPDPF. Furthermore, we confirmed that deficiency of HUWE1 enhances mitochondrial biogenesis and contributes to drug resistance in CRC cells. Taken together, our findings provide new insights into the role and molecular mechanisms of HUWE1 in regulating Wnt/β-catenin signaling.

## Results

### HUWE1 deficiency enhances characteristics of CSCs in CRC cells

**Fig. 1:**
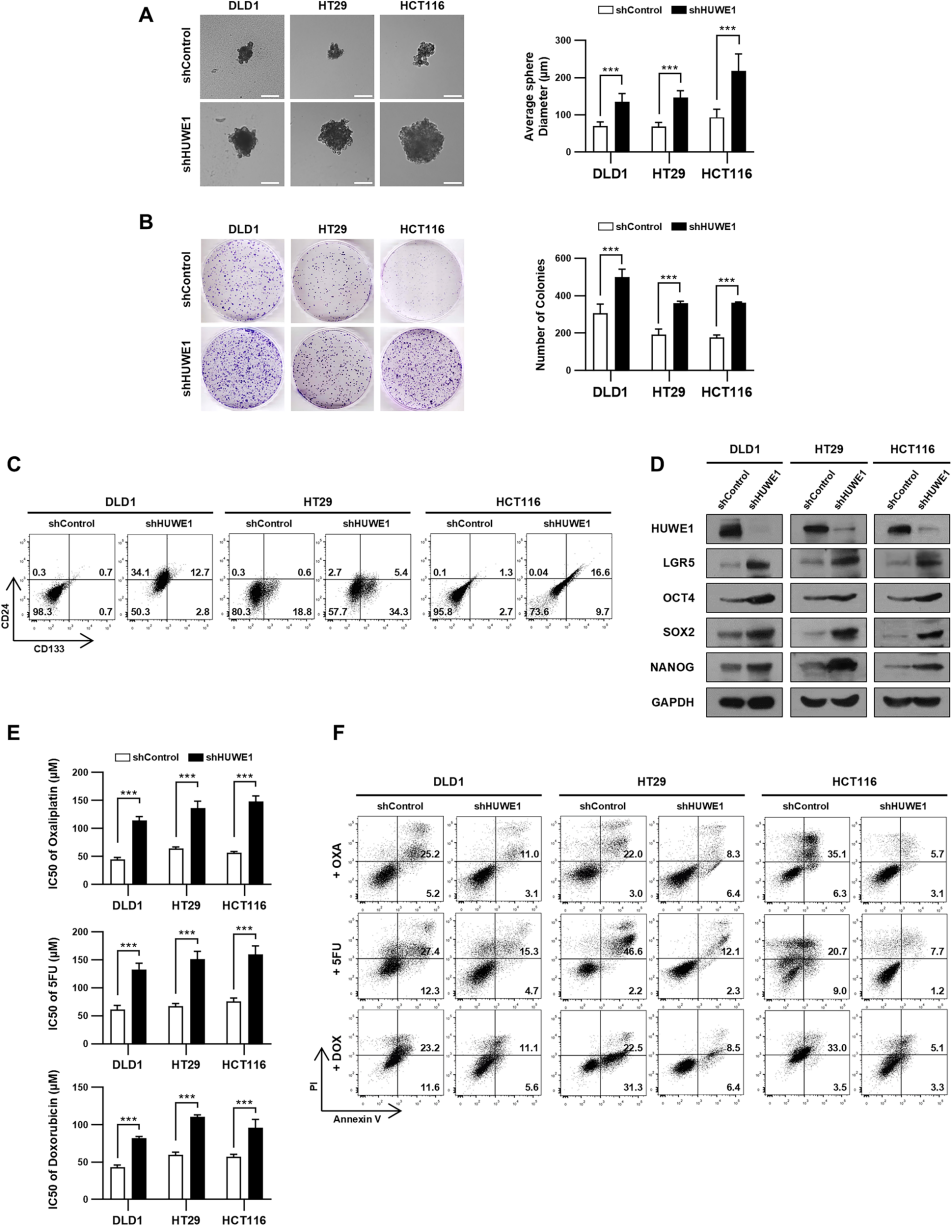
HUWE1 deficiency enhances characteristics of CSCs in CRC cells. **A** The tumor sphere-forming ability of DLD1, HT29, and HCT116 cells, with or without HUWE1 depletion, was examined. Scale bar, 100 µm. **B** Colony formation assay was performed in DLD1, HT29, and HCT116 cells with or without HUWE1 depletion. **C** Flow cytometry analysis was performed to assess the ratio of CD24+/CD133+ cells in DLD1, HT29, and HCT116 cells, with or without HUWE1 depletion. **D** Western blot analysis detected protein levels of LGR5, OCT4, SOX2, and NANOG in DLD1, HT29, and HCT116 cells, with or without HUWE1 depletion. **E** The IC50 values of oxaliplatin, 5FU, and doxorubicin in DLD1, HT29, and HCT116 cells, with or without HUWE1 depletion, were determined using the CCK-8 assay. **F** Apoptosis in DLD1, HT29, and HCT116 cells, with or without HUWE1 depletion, was assessed by flow cytometry using Annexin V-FITC and PI dual staining after treatment with 50 µM oxaliplatin, 5FU, or doxorubicin for 48 h. Data are presented as mean ± SEM. *P < 0.05, **P < 0.01, ***P < 0.001.

To examine the role of HUWE1 deficiency in promoting stemness in CRC, we suppressed HUWE1 expression in DLD1, HT29, and HCT116 cells using lentivirus-delivered shRNAs. Compared to control cells, HUWE1-deficient CRC cells exhibited increased sphere formation and colony formation capacity (Fig. 1A, B). FACS analysis further revealed a higher proportion of CD24^+^/CD133^+^ cells in HUWE1-deficient CRC cells (Fig. 1C, Supplementary Fig. 1A). CD24 and CD133 are well-known putative markers of the cancer stem cell population in CRC and are associated with aggressive tumor behavior [22–24]. Therefore, these results supported the notion that HUWE1-deficient CRC cells represent a cancer stem cell population. We also detected that expression of LGR5 and pluripotency transcription factors involved in stem cell maintenance was upregulated in HUWE1-deficient CRC cells (Fig. 1D, Supplementary Fig. 1B). Furthermore, HUWE1-deficient CRC cells displayed increased IC50 values for oxaliplatin, 5-fluorouracil (5FU), and doxorubicin, indicating enhanced chemoresistance (Fig. 1E). Consistently, these cells showed a significant reduction in apoptosis compared to control cells (Fig. 1F, Supplementary Fig. 1C). We also observed a greater shift of the PI+ population in the control group following doxorubicin treatment. This shift reflects increased intracellular accumulation of doxorubicin—which exhibits similar fluorescence similar to PI—rather than an actual increase in cell death. These findings suggest that HUWE1 loss enhances stemness and confers drug resistance in CRC cells.

### HUWE1 deficiency promotes cell proliferation, migration, and invasion in CRC cells

**Fig. 2:**
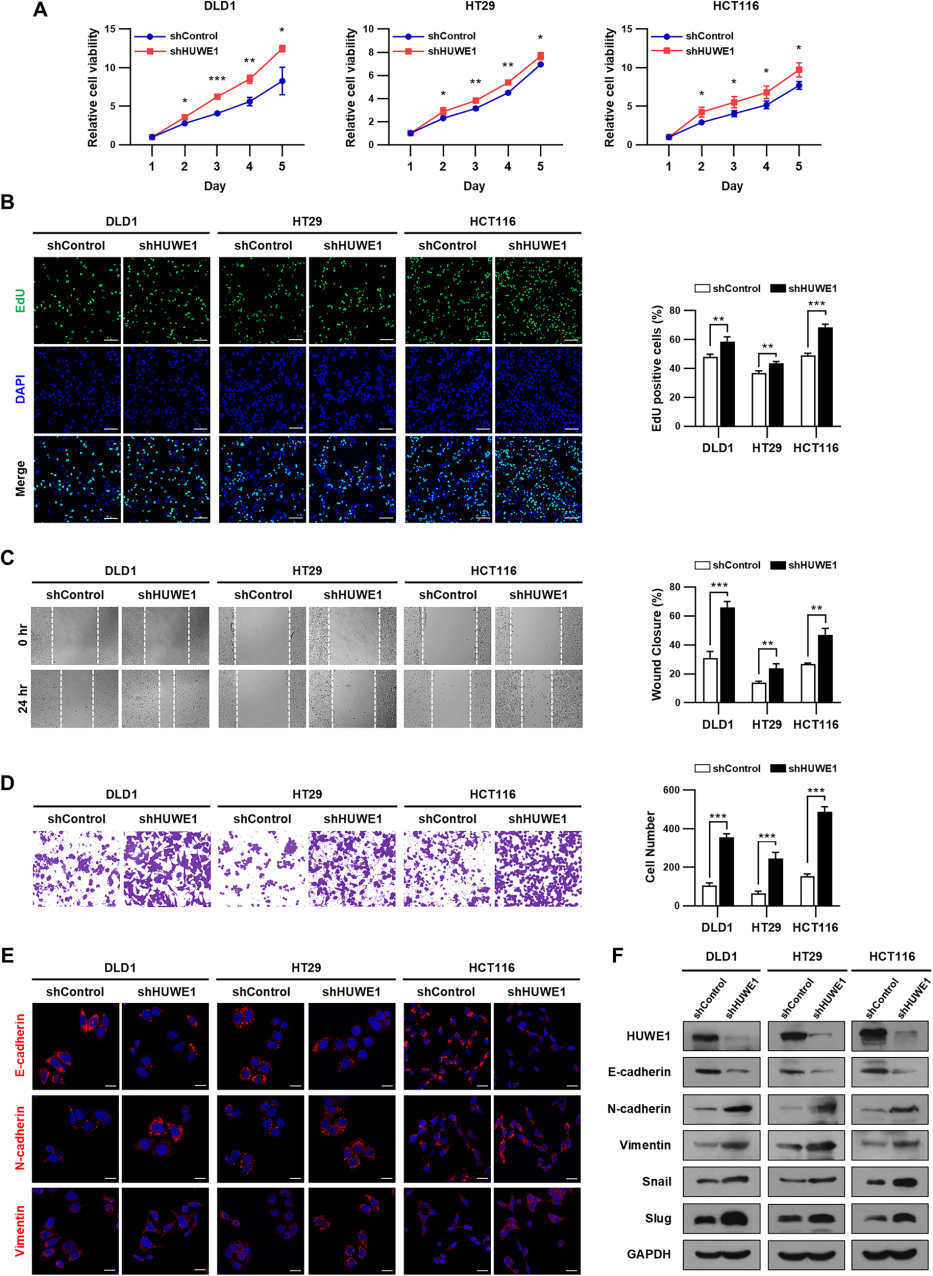
HUWE1 deficiency promotes cell proliferation, migration, and invasion in CRC cells. **A** The proliferation rate of DLD1, HT29, and HCT116 cells, with or without HUWE1 depletion, was examined using the CCK-8 assay. **B** EdU proliferation assay was performed in DLD1, HT29, and HCT116 cells, with or without HUWE1 depletion. Scale bar, 100 µm. **C** Migration capacity of DLD1, HT29, and HCT116, with or without HUWE1 depletion, was examined by wound healing assay. **D** Transwell invasion assay was performed in DLD1, HT29, and HCT116 cells, with or without HUWE1 depletion. **E** Immunofluorescence analysis was performed to examine the expression of E-cadherin, N-cadherin, and Vimentin in DLD1, HT29, and HCT116 cells, with or without HUWE1 depletion. Scale bar, 20 µm. **F** Western blot analysis of DLD1, HT29, and HCT116 cells, with or without HUWE1 depletion, was performed with the indicated antibodies. Data are presented as mean ± SEM. *P < 0.05, **P < 0.01, ***P < 0.001.

To evaluate whether HUWE1 depletion affects cell growth in CRC cells, we performed a CCK-8 assay and found that suppression of HUWE1 significantly increased cell proliferation (Fig. 2A). Consistently, the number of EdU-positive cells was notably high in HUWE1-deficient CRC cells compared to the control cells (Fig. 2B). Next, we investigated the effect of HUWE1 depletion on the migration and invasion of CRC cells. HUWE1-deficient CRC cells exhibited faster wound closure, indicating an increased migration rate (Fig. 2C). Similarly, a transwell invasion assay revealed a higher number of invaded cells in HUWE1-deficient CRC cells compared to controls (Fig. 2D). We further examined the role of HUWE1 depletion in regulating epithelial-mesenchymal transition (EMT) markers using immunofluorescence analysis. Compared to control cells, HUWE1-deficient CRC cells displayed decreased E-cadherin expression and increased levels of N-cadherin and Vimentin (Fig. 2E). Consistent with these findings, western blot analysis revealed reduced E-cadherin expression alongside increased expression of N-cadherin, Vimentin, Snail, and Slug in HUWE1-deficient CRC cells (Fig. 2F, Supplementary Fig. 2A). In addition, cellular morphological analysis using Phalloidin-iFluor 647 Reagent revealed that control CRC cells exhibited a typical cuboidal morphology, whereas HUWE1-deficient CRC cells displayed a more elongated and mesenchymal-like phenotype (Supplementary Fig. 2B). These results suggest that loss of HUWE1 promotes cell proliferation, migration, invasion, and EMT progression in CRC cells.

### HUWE1 regulates Wnt/β-catenin signaling under conditions of β-catenin destruction complex dysfunction

**Fig. 3:**
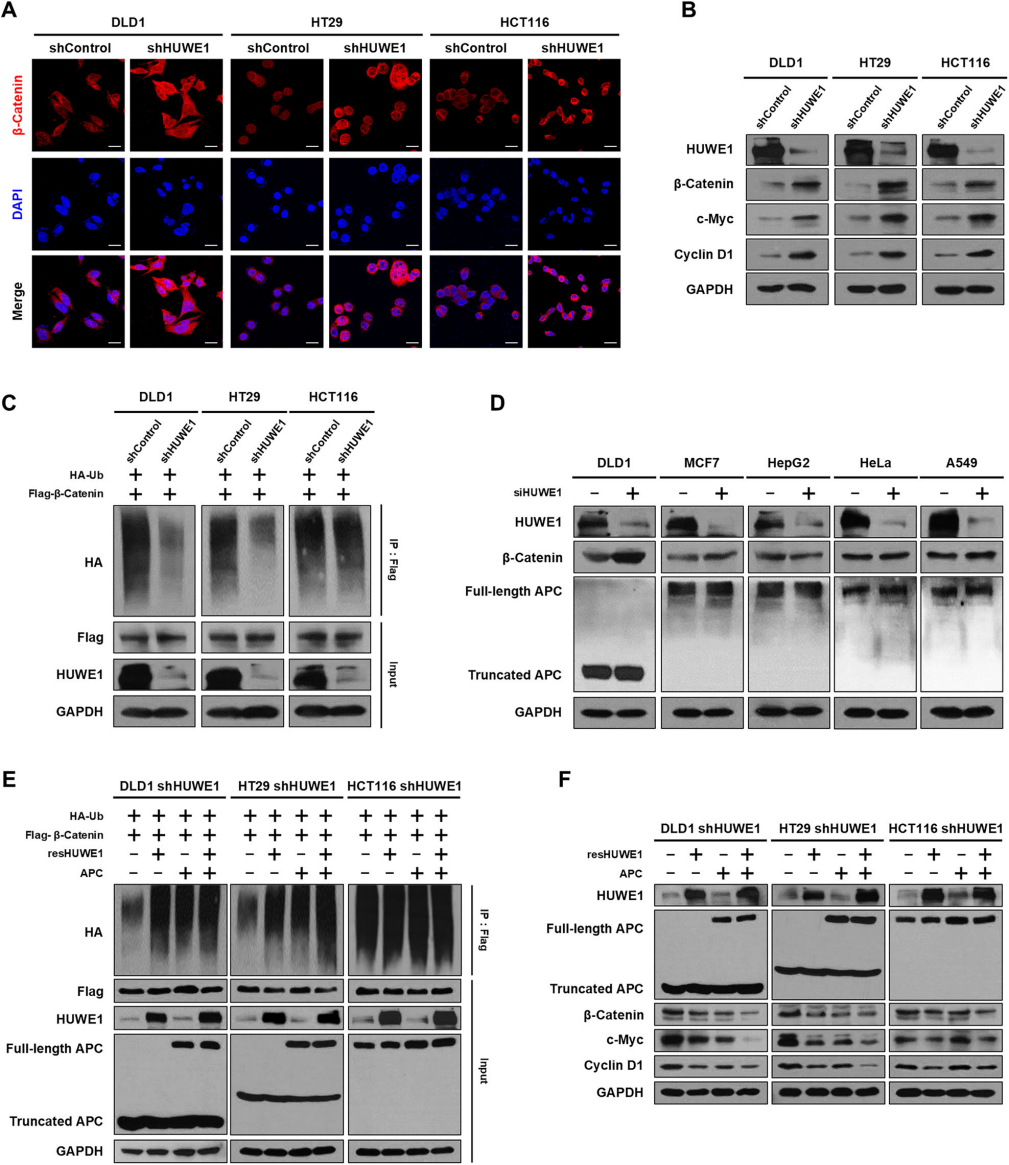
HUWE1 regulates Wnt/β-catenin signaling under conditions of β-catenin destruction complex dysfunction. **A** Immunofluorescence analysis was performed to detect the expression of β-catenin in the nucleus and cytoplasm of DLD1, HT29, and HCT116 cells, with or without HUWE1 depletion. Scale bar, 20 µm. **B** Western blot analysis detected the expression of β-catenin, c-Myc, and Cyclin D1 in DLD1, HT29, and HCT116 cells, with or without HUWE1 depletion. **C** DLD1, HT29, and HCT116 cells, with or without HUWE1 depletion, were transfected with Flag-β-catenin and HA-Ub plasmids. The degree of ubiquitination of β-catenin was analyzed using an immunoprecipitation assay. **D** DLD1, MCF7, HepG2, HeLa, and A549 cells were transfected with *HUWE1* siRNA, and then the expression level of β-catenin was detected by western blot analysis. **E** DLD1, HT29, and HCT116 cells with HUWE1 depletion were transfected with plasmids encoding for Flag-β-catenin, resHUWE1, full-length APC, and HA-Ub. The degree of ubiquitination of β-catenin was analyzed using an immunoprecipitation assay. **F** DLD1, HT29, and HCT116 cells with HUWE1 depletion were transfected with plasmids encoding for resHUWE1 and full-length APC, and then expression levels of β-catenin, c-Myc, and Cyclin D1 were detected by western blot analysis. Data are presented as mean ± SEM. *P < 0.05, **P < 0.01, ***P < 0.001.

To investigate whether HUWE1 depletion affects the regulation of Wnt/β-catenin signaling, we analyzed β-catenin expression levels in HUWE1-deficient CRC cells. Immunofluorescence analysis revealed a significant accumulation of β-catenin in both the nucleus and cytoplasm of HUWE1-deficient CRC cells (Fig. 3A). Consistently, western blot analysis demonstrated increased expression of Wnt/β-catenin downstream target genes, including c-Myc and Cyclin D1, in HUWE1-deficient CRC cells (Fig. 3B, Supplementary Fig. 3A).

To determine whether HUWE1 depletion impacts β-catenin ubiquitination, we performed a ubiquitination assay. HUWE1-deficient CRC cells were co-transfected with plasmids containing Flag-β-catenin and HA-Ub, followed by co-immunoprecipitation (Co-IP) to detect ubiquitinated β-catenin. The results showed a reduction in β-catenin ubiquitination in HUWE1-deficient DLD1 and HT29 cells compared to control cells (Fig. 3C). However, HUWE1-deficient HCT116 cells, which possess wild-type APC, exhibited no significant changes in β-catenin ubiquitination levels. Previous studies have shown that HUWE1 loss results in excessive β-catenin accumulation specifically in the absence of functional APC [15]. Given the high prevalence of *APC* mutations in CRC (Supplementary Fig. 3B), we examined whether the HUWE1 depletion similarly impacts β-catenin accumulation in other types of cancer cells with the wild-type APC. In DLD1 cells with truncated APC, HUWE1 suppression resulted in a substantial accumulation of β-catenin, a phenomenon not observed in other cancer cells with intact APC (Fig. 3D, Supplementary Fig. 3C). Furthermore, in RKO cells, which have an intact Wnt/β-catenin signaling pathway, dual inhibition of HUWE1 and APC led to greater β-catenin accumulation compared to APC inhibition alone (Supplementary Fig. 3D). Collectively, these findings indicate that HUWE1 facilitates β-catenin degradation, particularly in the context of APC dysfunction, highlighting its role as a key regulator of Wnt/β-catenin signaling in CRC.

To confirm the specificity of HUWE1 knockdown and perform rescue experiments, we generated a HUWE1 construct (resHUWE1) resistant to the target nucleotide sequence of the HUWE1 shRNA (Supplementary Fig. 3E). Using the construct, we assessed the degradation of β-catenin upon overexpression of resHUWE1 or full-length APC in HUWE1-deficient CRC cells. In HUWE1-deficient DLD1 and HT29 cells, overexpression of resHUWE1 or full-length APC significantly increased β-catenin ubiquitination (Fig. 3E). However, in HUWE1-deficient HCT116 cells (which harbor a serine 45 deletion in β-catenin), no notable differences in β-catenin ubiquitination were observed.

Interestingly, overexpression of resHUWE1 reduced the expression of endogenous β-catenin and its downstream target proteins (c-Myc and Cyclin D1) across HUWE1-deficient DLD1, HT29, and HCT116 cells (Fig. 3F, Supplementary Fig. 3F). In contrast, overexpression of full-length APC failed to reduce β-catenin levels and downstream target protein expression in HUWE1-deficient HCT116 cells (Fig. 3F, Supplementary Fig. 3F). Moreover, overexpression of the catalytically inactive resHUWE1 C4341S mutant also failed to decrease β-catenin and its downstream target proteins, clearly demonstrating that the degradation of β-catenin is dependent on the E3 ligase activity of HUWE1 (Supplementary Fig. 4). These results suggest that HUWE1 specifically regulates β-catenin degradation and Wnt/β-catenin signaling under conditions of β-catenin destruction complex dysfunction, particularly when APC function is impaired or β-catenin mutations disrupt the normal degradation pathway.

### HUWE1 enhances the interaction with β-catenin under conditions of β-catenin destruction complex dysfunction

**Fig. 4:**
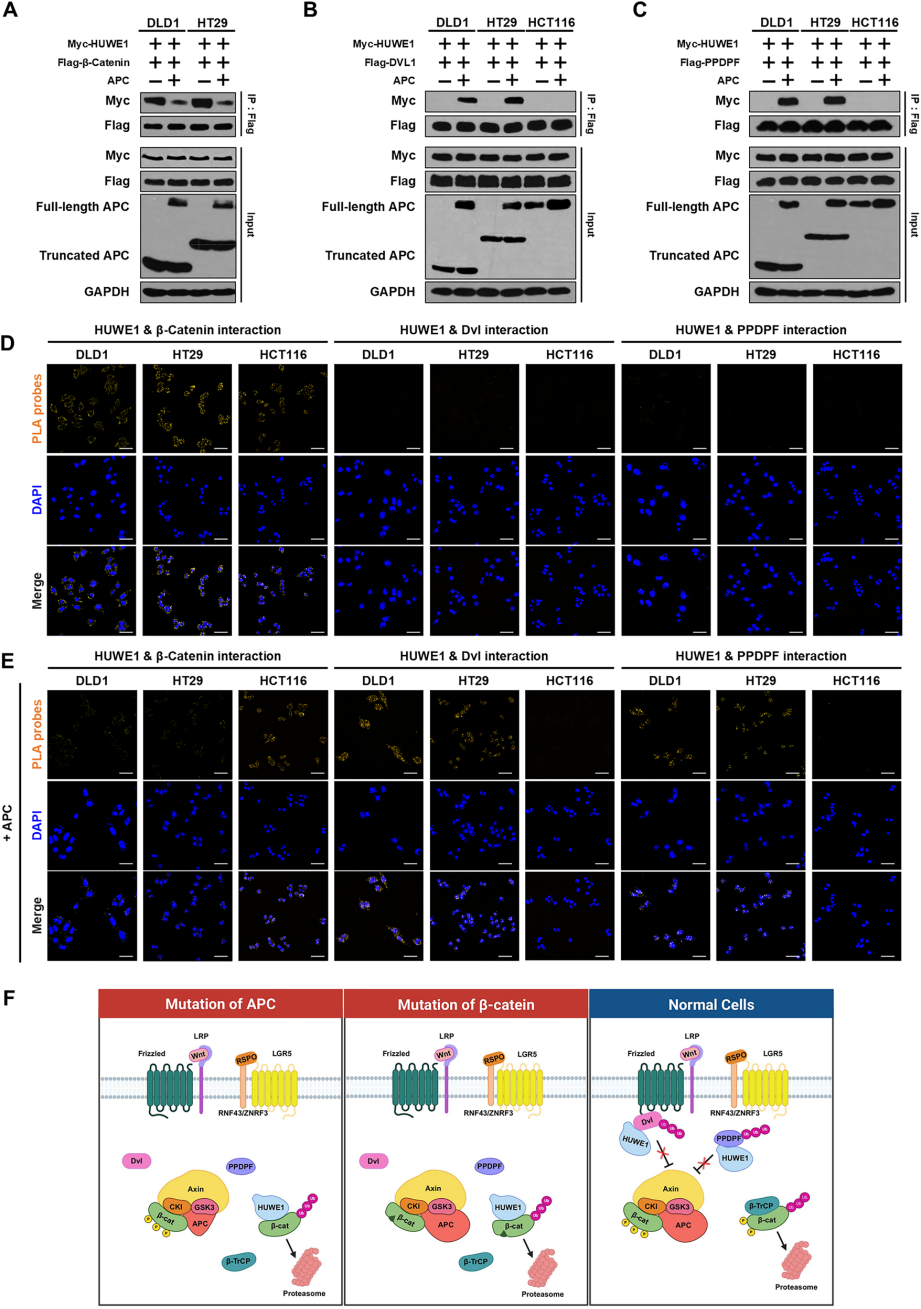
HUWE1 enhances its interaction with β-catenin under conditions of β-catenin destruction complex dysfunction. **A** Comparison of the interaction between HUWE1 and β-catenin in DLD1 and HT29 cells transfected with control plasmid or full-length APC plasmid using Co-immunoprecipitation assay. **B**, **C** Comparison of the interaction between HUWE1 and DVL1 or PPDPF in DLD1, HT29, and HCT116 cells transfected with control or full-length APC plasmids using Co-immunoprecipitation assay. **D**, **E** PLA signal was observed in DLD1, HT29, and HCT116 cells transfected with or without full-length APC plasmid by confocal microscopy. Scale bar, 50 µm. **F** Schematic diagram illustrating a model of HUWE1 protein interactions in the Wnt/β-catenin signaling pathway, depending on the activation status of the β-catenin destruction complex.

The results described above suggest that HUWE1 plays a role in β-catenin degradation, similarly to the β-catenin destruction complex. However, it is unclear whether HUWE1 directly degrades β-catenin when the β-catenin destruction complex is functional. To address this, we overexpressed full-length APC in CRC cells and evaluated the interaction between HUWE1 and β-catenin using Co-IP. Overexpression of full-length APC in DLD1 and HT29 cells significantly reduced the interaction between HUWE1 and β-catenin (Fig. 4A). Interestingly, overexpression of full-length APC in DLD1 and HT29 cells displayed the interaction between HUWE1 and upstream Wnt/β-catenin signaling proteins, such as DVL1 and PPDPF, whereas no such interaction was observed in HCT116 cells (Fig. 4B, C). These findings were further validated by proximity ligation assay (PLA). We detected amplified PLA signals in DLD1, HT29, and HCT116 cells labeled with HUWE1 and β-catenin primary antibodies, but no signals were observed in cells labeled with HUWE1 and Dvl or HUWE1 and PPDPF primary antibodies (Fig. 4D). However, overexpression of full-length APC reduced HUWE1-β-catenin PLA signals only in DLD1 and HT29 cells, but not in HCT116 cells, while signals corresponding to HUWE1-DVL1 and HUWE1-PPDPF interactions became apparent (Fig. 4E). Furthermore, in RKO cells, inhibition of APC led to an increased interaction between HUWE1 and β-catenin, while the interactions between HUWE1 and Dvl, as well as HUWE1 and PPDPF, were decreased (Supplementary Fig. 5A–D). These results suggest that in the Wnt-on hyperactivation state, HUWE1 primarily facilitates β-catenin degradation under conditions of β-catenin destruction complex dysfunction caused by mutations in APC (as in DLD1 and HT29 cells) or β-catenin (as in HCT116 cells). On the other hand, in the conditions where the β-catenin destruction complex remains functional (as in RKO cells), HUWE1 interacts with Dvl or PPDPF to fine-tune Wnt/β-catenin signaling (Fig. 4F).

### HUWE1 regulates cell proliferation, migration, invasion, and stemness in CRC cells

**Fig. 5:**
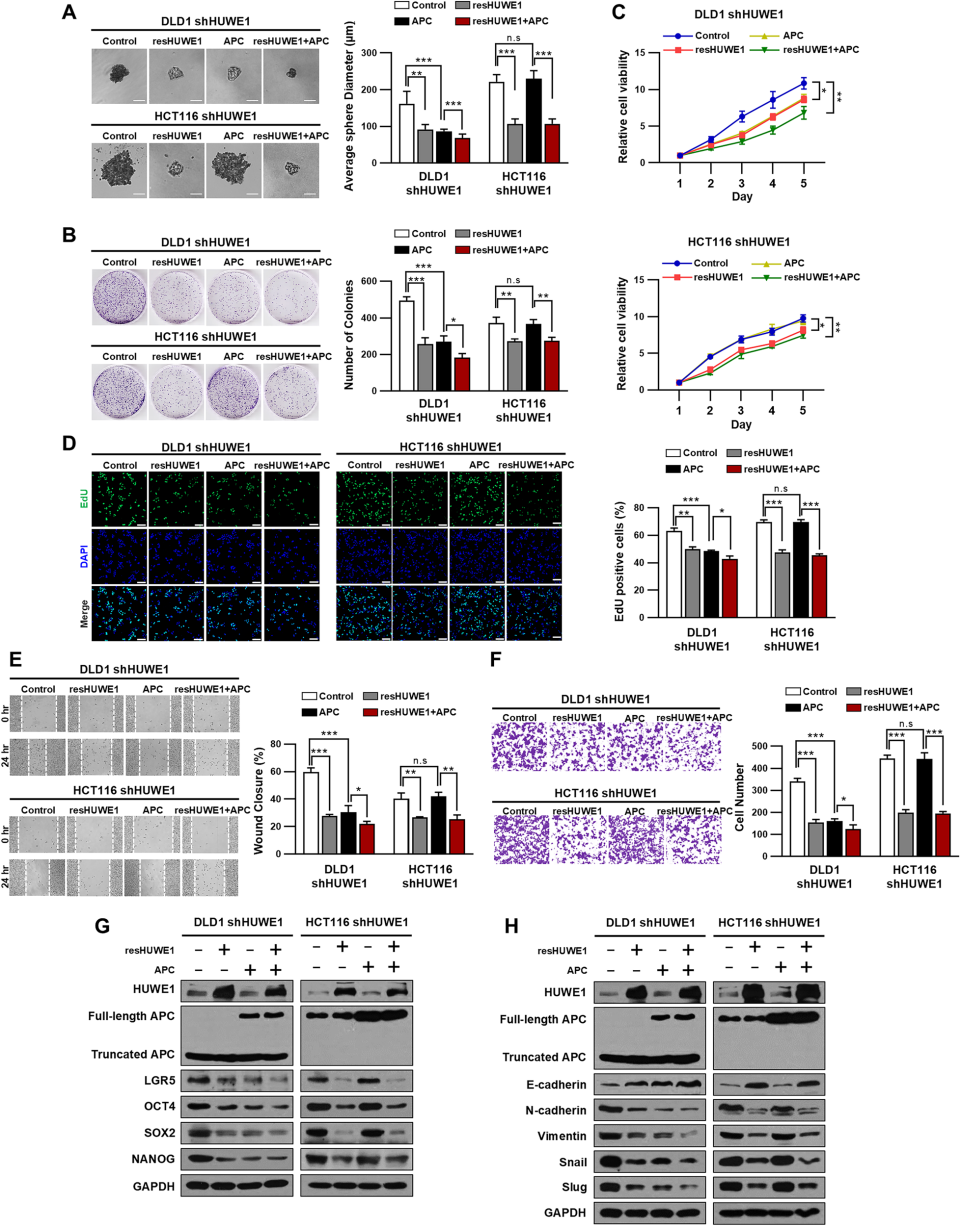
HUWE1 regulates cell proliferation, migration, invasion, and stemness in CRC cells. DLD1 and HCT116 cells, with or without HUWE1 depletion, were transfected with expression plasmids for resHUWE1, full-length APC, or both. **A** Tumor sphere-forming ability of the indicated cells was examined. Scale bar, 100 µm. **B** Colony formation assay was performed using the indicated cells. **C** The proliferation rate of the indicated cells was examined using the CCK-8 assay. **D** EdU proliferation assay was performed using the indicated cells. Scale bar, 100 µm. **E** Migration capacity of the indicated cells was examined by wound healing assay. **F** Transwell invasion assay was performed using the indicated cells. **G**, **H** Western blot analysis of the indicated cells was performed using the specified antibodies. Data are presented as mean ± SEM. *P < 0.05, **P < 0.01, ***P < 0.001.

Next, we investigated whether the overexpression of resHUWE1 could reverse the enhanced cell proliferation, migration, invasion, and stemness induced by HUWE1 depletion in CRC cells. Overexpression of resHUWE1 significantly reduced the sphere formation efficiency and colony formation capacity in HUWE1-deficient DLD1 and HCT116 cells (Fig. 5A, B). In addition, resHUWE1 overexpression inhibited cell proliferation in HUWE1-deficient DLD1 and HCT116 cells (Fig. 5C). Consistently, we observed a decrease in EdU-positive cells upon resHUWE1 overexpression, indicating reduced DNA synthesis and proliferation (Fig. 5D). Furthermore, resHUWE1 overexpression reduced both cell migration and invasion rates in HUWE1-deficient DLD1 and HCT116 cells (Fig. 5E, F). Western blot analysis revealed that resHUWE1 overexpression reduces the expression of stemness-associated markers, including LGR5, OCT4, SOX2, and NANOG, in HUWE1-deficient DLD1 and HCT116 cells (Fig. 5G, Supplementary Fig. 6A). Overexpression of resHUWE1 also led to an increase in the expression of E-cadherin and decreased expression of N-cadherin, Vimentin, Snail, and Slug in HUWE1-deficient CRC cells (Fig. 5H, Supplementary Fig. 6B). Interestingly, while full-length APC overexpression effectively reduced cell proliferation, migration, invasion, and stemness in HUWE1-deficient DLD1 cells, it failed to produce the same effect in HUWE1-deficient HCT116 cells. Moreover, in HUWE1-deficient DLD1 cells, these effects were increased when both resHUWE1 and APC were overexpressed simultaneously compared to APC overexpression alone. We also demonstrated that overexpression of wild-type resHUWE1 reduced the expression of stemness-associated markers in HUWE1-deficient DLD1 and HCT116 cells, whereas the catalytically inactive resHUWE1 C4341S mutant showed no such effect (Supplementary Fig. 7). These findings collectively indicate that HUWE1 regulates Wnt/β-catenin signaling in CRC in coordination with the β-catenin destruction complex, playing a crucial role in controlling cell proliferation, migration, invasion, and stemness.

### HUWE1 deficiency induces drug resistance by promoting mitochondrial biogenesis in CRC cells

**Fig. 6:**
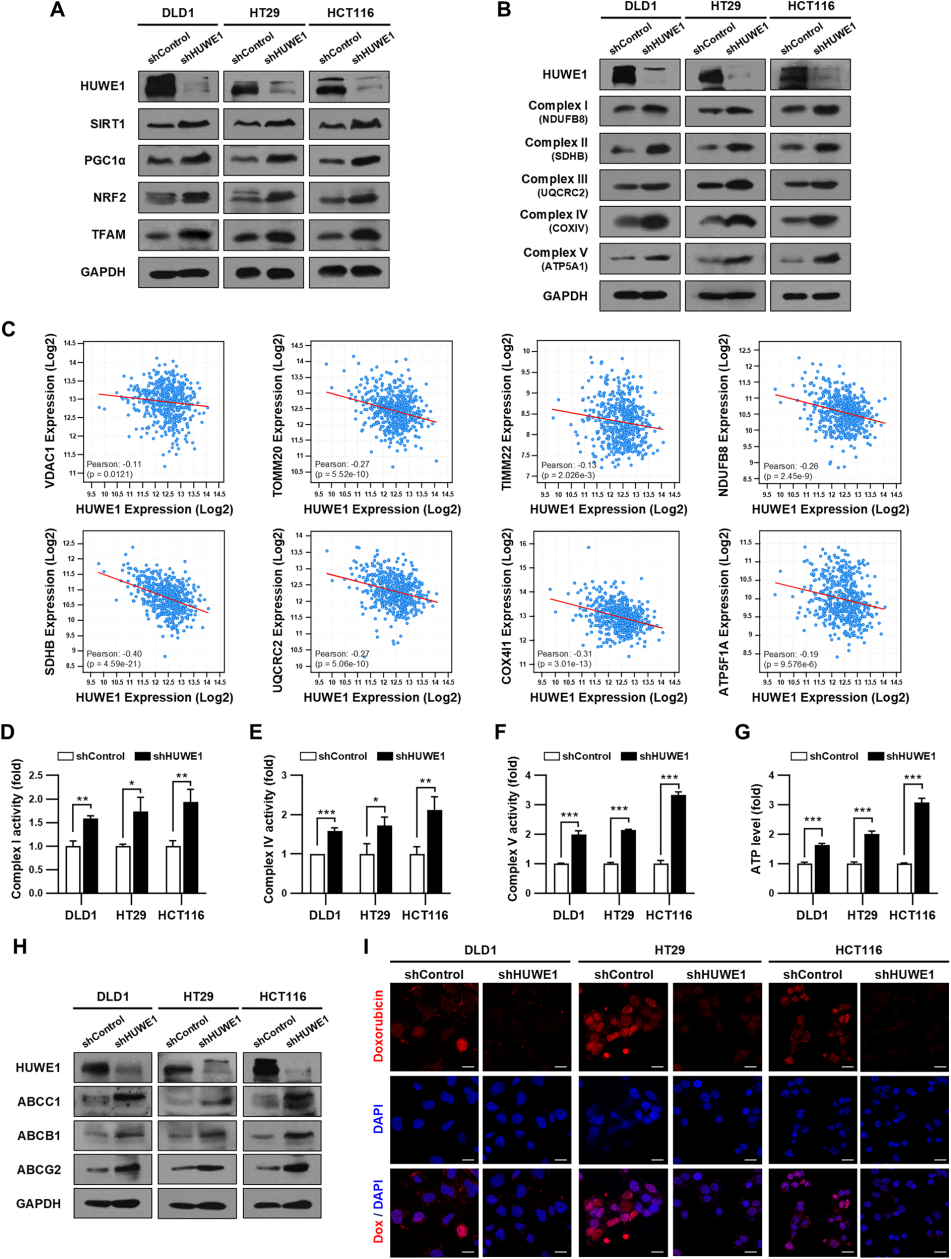
HUWE1 deficiency induces drug resistance by promoting mitochondrial biogenesis in CRC cells. **A**, **B** Western blot analysis of DLD1, HT29, and HCT116 cells, with or without HUWE1 depletion, was performed with the indicated antibodies. **C** Negative correlations were observed between the expression levels of HUWE1 and various mitochondrial proteins (VDAC1, TOMM20, TIMM22, NDUFB8, SDHB, UQCRC2, COX4, and ATP5A1) in CRC. **D**–**F** Measurement of mitochondrial ETC complex activities in DLD1, HT29, and HCT116 cells, with or without HUWE1 depletion. **G** Measurement of ATP level in DLD1, HT29, and HCT116 cells, with or without HUWE1 depletion. **H** Western blot analysis detected the expression of ABCC1, ABCB1, and ABCG2 in DLD1, HT29, and HCT116 cells, with or without HUWE1 depletion. **I** DLD1, HT29, and HCT116 cells, with or without HUWE1 depletion, were incubated with 10 µM doxorubicin for 24 h. Cells were then stained with DAPI and observed by confocal microscopy for doxorubicin fluorescence. Data are presented as mean ± SEM. *P < 0.05, **P < 0.01, ***P < 0.001.

Next, we investigated whether mitochondrial biogenesis is upregulated by HUWE1 depletion in CRC cells. We observed an increase in the expression of key transcriptional regulators involved in mitochondrial biogenesis, such as SIRT1, PGC1α, NRF2, and TFAM, in HUWE1-deficient CRC cells (Fig. 6A, Supplementary Fig. 8A). Additionally, the expression of mitochondrial electron transfer chain (ETC) complex subunit proteins, including NDUFB8, SDHB, UQCRC2, COXIV, and ATP5A1, was also elevated in HUWE1-deficient CRC cells (Fig. 6B, Supplementary Fig. 8B). To further validate these results, we analyzed the TCGA CRC dataset and found that HUWE1 expression was negatively correlated with the expression levels of most mitochondrial proteins (Fig. 6C). Moreover, we observed that the ETC activity and ATP production were significantly enhanced in HUWE1-deficient CRC cells compared to the control cells (Fig. 6D–G). These results suggest that depletion of HUWE1 promotes mitochondrial biogenesis, inducing ETC activity, and consequently increasing ATP production in CRC cells.

Additionally, we observed elevated expression of ABC transporter proteins in HUWE1-deficient CRC cells (Fig. 6H, Supplementary Fig. 8C). To assess the impact of HUWE1 depletion on drug efflux effects, we measured the release of doxorubicin over a 24 h period. While control cells retained doxorubicin, it was nearly absent in the HUWE1-deficient CRC cells (Fig. 6I, Supplementary Fig. 8D). In addition, pharmacological inhibition of mitochondrial ATP production with oligomycin increased drug sensitivity in HUWE1-deficient CRC cells (Supplementary Fig. 9A, B). Similarly, inhibiting ABC transporter activity with tariquidar also enhanced drug sensitivity (Supplementary Fig. 9C). Collectively, these findings suggest that loss of HUWE1 in CRC cells leads to increased ATP production, which likely supports the overexpressed ABC transporter proteins, contributing to drug resistance.

### HUWE1 increases drug sensitivity through downregulation of mitochondrial biogenesis in CRC cells

**Fig. 7:**
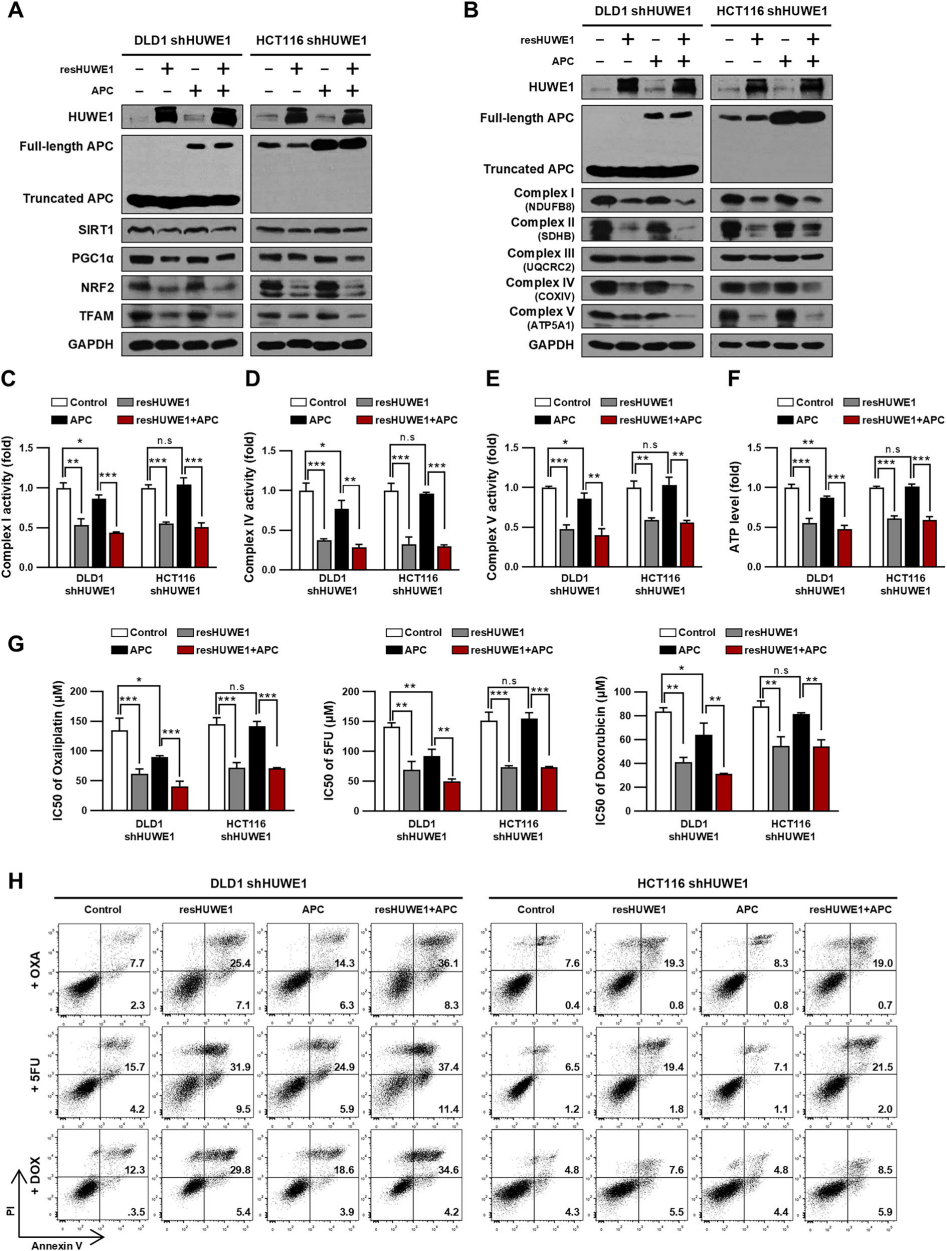
HUWE1 enhances drug sensitivity by downregulating mitochondrial biogenesis in CRC cells. DLD1 and HCT116 cells, with or without HUWE1 depletion, were transfected with the expression plasmid for resHUWE1, full-length APC, or both. **A**, **B** Western blot analysis of the indicated cells was performed using the specified antibodies. **C**–**E** Measurement of mitochondrial ETC complex activities in the indicated cells. **F** Measurement of ATP level in the indicated cells. **G** The IC50 of oxaliplatin, 5FU, and doxorubicin in the indicated cells was determined using the CCK-8 assay. **H** Apoptosis in the indicated cells was observed using flow cytometry analysis with Annexin V-FITC and PI dual staining after treatment with 50 µM oxaliplatin, 5FU, or doxorubicin for 48 h. Data are presented as mean ± SEM. *P < 0.05, **P < 0.01, ***P < 0.001.

To investigate whether overexpression of resHUWE1 could modulate mitochondrial biogenesis enhanced by HUWE1 depletion in CRC cells. We found that resHUWE1 overexpression significantly reduced the expression of proteins involved in mitochondrial biogenesis and ETC complex subunit in both HUWE1-deficient DLD1 and HCT116 cells, whereas overexpression of full-length APC only slightly decreased the expression of these proteins in HUWE1-deficient DLD1 cells (Fig. 7A, B, Supplementary Fig. 10A, B). Moreover, resHUWE1 overexpression led to a decrease in ETC activity and ATP production in both HUWE1-deficient DLD1 and HCT116 cells, whereas overexpression of full-length APC had a mild or no effect on these in HUWE1-deficient DLD1 or HCT116 cells, respectively (Fig. 7C–F). These results indicate that HUWE1 plays a crucial role in regulating mitochondrial biogenesis in CRC cells.

To determine whether drug resistance induced by HUWE1 depletion in CRC cells could be reversed upon HUWE1 restoration, we overexpressed resHUWE1 in HUWE1-deficient DLD1 and HCT116 cells and assessed their drug sensitivity. We observed a decrease in IC50 values for oxaliplatin, 5FU, and doxorubicin in both HUWE1-deficient DLD1 and HCT116 cells upon resHUWE1 overexpression (Fig. 7G). Furthermore, resHUWE1 overexpression significantly enhanced apoptosis in both HUWE1-deficient DLD1 and HCT116 cells (Fig. 7H, Supplementary Fig. 10C). In contrast, full-length APC overexpression increased drug sensitivity and apoptosis only in HUWE1-deficient DLD1 cells, and the effect was less pronounced than with resHUWE1 overexpression. Taken together, these results suggest that HUWE1 contributes to increased drug sensitivity by regulating not only Wnt/β-catenin signaling but also mitochondrial biogenesis in CRC cells.

## Discussion

APC plays a crucial role in the assembly and reorientation of the β-catenin destruction complex in cells [25]. As shown in Supplementary Fig. 3B, a significant proportion of CRCs harbors *APC* mutations. These mutations disrupt the ubiquitination and subsequent proteasomal degradation of β-catenin, leading to aberrant activation of the Wnt/β-catenin signaling pathway [26].

HUWE1 has been reported to negatively regulate several proteins involved in Wnt/β-catenin signaling [13–15]. In this study, we investigated the mechanism by which HUWE1 regulates the hyperactivated Wnt/β-catenin signaling in CRC. Our findings indicate that under conditions of excessive β-catenin accumulation due to *APC* mutations, HUWE1 primarily interacts with β-catenin to promote its degradation. Although we did not compare the ubiquitination levels of β-catenin between the HUWE1 wild-type and HUWE1 C4341S overexpression, the PLA assay confirmed an interaction between HUWE1 and β-catenin, and the failure of HUWE1 C4341S overexpression to promote β-catenin degradation suggests that HUWE1 ubiquitinates β-catenin. However, in cells with normal APC, such as RKO cells, HUWE1 is less involved in direct regulation of β-catenin and instead interacts primarily with Dvl or PPDPF proteins. Interestingly, in HCT116 cells, which possess normal APC but harbor a β-catenin allele with a mutation at the GSK3β phosphorylation site [27, 28], HUWE1 predominantly interacts with β-catenin. In HUWE1-deficient HCT116 cells, although normal APC is present, β-catenin accumulation, stemness, and EMT were upregulated compared to the control cells. Furthermore, recovery experiments demonstrated that in HUWE1-deficient DLD1 and HT29 cells, reintroduction of HUWE1 or APC reduced CSCs characteristics. In contrast, in HUWE1-deficient HCT116 cells, only the rescue of HUWE1 was effective. These results suggest that the phosphorylation of β-catenin by the β-catenin destruction complex may influence its interaction with HUWE1. When β-catenin is properly phosphorylated by this destruction complex, it is typically targeted for ubiquitination by Beta-Transducin Repeats-Containing Proteins (β-TrCP). However, when the β-catenin destruction complex fails to phosphorylate β-catenin, ubiquitination by β-TrCP is impaired, resulting in accumulation of β-catenin within the cell. In this context, since the interaction between HUWE1 and β-catenin is independent of phosphorylation [15], HUWE1 can interact and degrade the accumulated β-catenin (Fig. 4F).

**Fig. 8:**
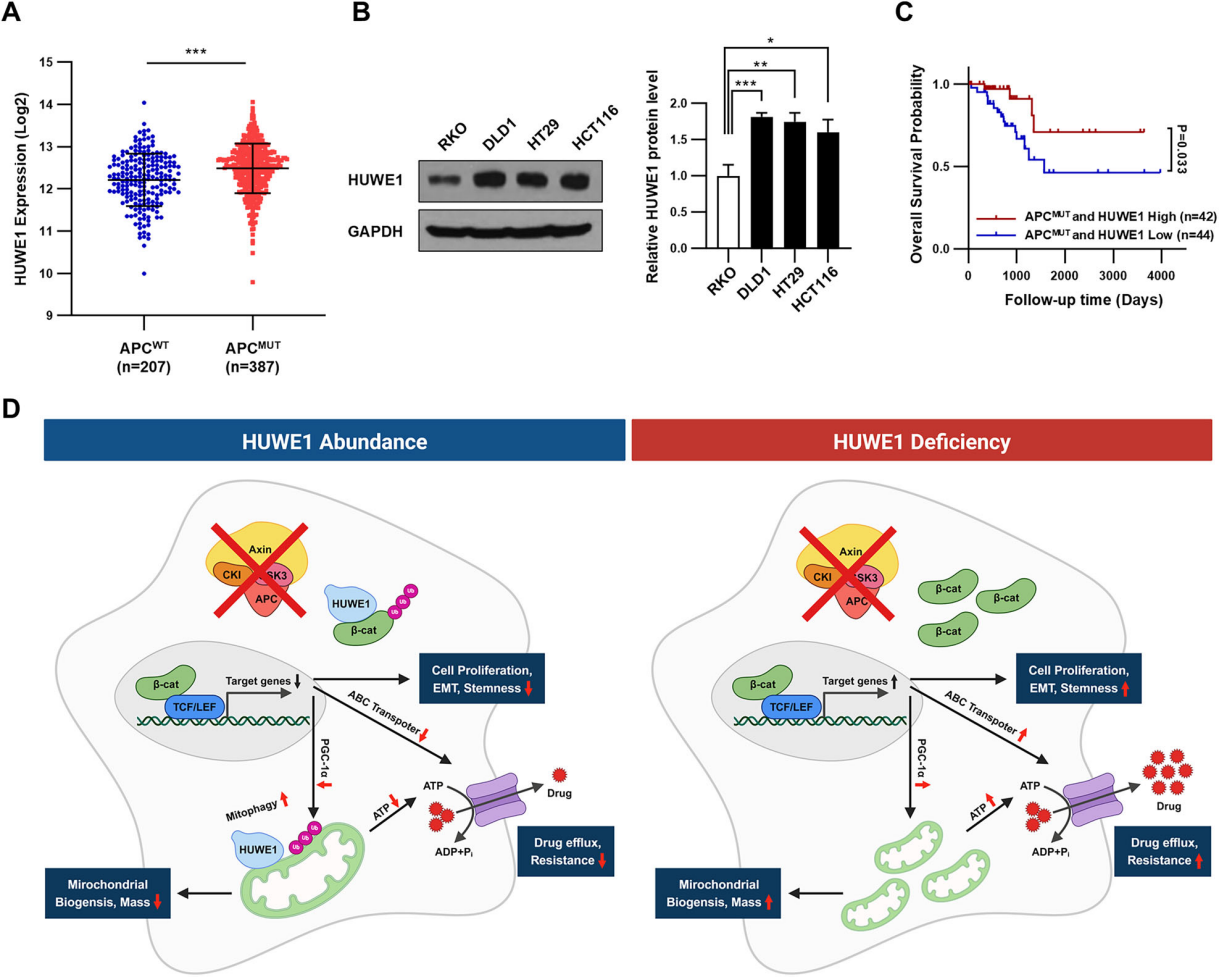
Deficiency of HUWE1 promotes stemness and drug resistance under conditions of β-catenin destruction complex dysfunction in CRC. **A** Relationship between *APC* mutations and *HUWE1* mRNA levels in 594 CRC samples. The data were extracted from the cBioPortal database (https://www.cbioportal.org/). **B** Comparison of HUWE1 protein levels quantified by Western blot analysis in RKO, DLD1, HT29, and HCT116 cells. **C** Kaplan–Meier plot of overall survival for CRC patients with deleterious *APC* mutations, stratified by HUWE1 expression levels, analyzed using the log-rank test. **D** Schematic diagram summarizing the findings of this study. Data are presented as mean ± SEM. *P < 0.05, **P < 0.01, ***P < 0.001.

Previous studies have reported that HUWE1 is overexpressed in CRC tumor tissues [29, 30]. Moreover, its expression was found to be higher in the CRC cases with mutant *APC* (Fig. 8A). Consistent with this, we observed lower expression of HUWE1 in RKO cells compared to DLD1, HT29, and HCT116 cells (Fig. 8B). These observations suggest that in cells exhibiting β-catenin stabilization, enhanced cancer stemness, and drug resistance—arising from dysfunction of the β-catenin destruction complex—HUWE1 expression may be induced as a compensatory mechanism to counteract the excessive activation of the Wnt/β-catenin signaling pathway. However, inhibition of APC in RKO cells did not result in an increased HUWE1 expression. Although previous reports have suggested a potential link between HUWE1 mutations and the acquisition of mutations in APC or other Wnt signaling components, this relationship remains unclear due to the limited number of reported cases [15]. While we cannot conclude whether HUWE1 loss alone is sufficient to regulate Wnt/β-catenin signaling in CRC, our findings demonstrate that the simultaneous loss of both the β-catenin destruction complex and HUWE1 accelerates the transition of cancer cells to CSC-like states.

In our previous study, we demonstrated that HUWE1 enhances oxaliplatin sensitivity in CRC by promoting the degradation of TOMM20 [21]. However, the exact mechanism underlying oxaliplatin resistance due to HUWE1 deficiency remained unclear, partly because the observations were made in mixed cell populations. In this study, we confirmed that HUWE1 loss alone can induce drug resistance in CRC and identified the underlying cause.

Mitochondrial mass and quality are tightly regulated by two essential and opposing mechanisms—mitochondrial biogenesis and mitophagy—in response to cellular energy demands and various cellular and environmental signals [31]. HUWE1 is recruited to mitochondria by AMBRA1 and plays a role in promoting mitophagy [21, 32, 33]. Therefore, HUWE1 deficiency may impair mitophagy, leading to excessive mitochondrial biogenesis and increased mitochondrial mass [34, 35]. This mitochondrial imbalance results in elevated ATP production, which enhances ABC transporter-mediated drug efflux, contributing to chemotherapy resistance [20]. Indeed, our results demonstrated that excessive mitochondrial biogenesis in HUWE1-deficient CRC cells significantly increased ATP production, facilitating drug efflux via ABC transporters. Furthermore, we found that restoring HUWE1 in HUWE1-deficient DLD1 cells was more effective in reducing excessive mitochondrial biogenesis and improving drug sensitivity than restoring APC function. Therefore, gene therapy targeting HUWE1 could be a potential therapeutic approach to overcome drug resistance in CRC induced by HUWE1 depletion. However, the large size of HUWE1 may present a barrier to effective gene therapy [36]. Therefore, it is necessary to either identify molecules that enhance HUWE1-mediated ubiquitination or to engineer truncated versions of HUWE1 by removing non-essential domains, thereby generating functionally active shorter fragments. Taken together, we emphasize the critical role of HUWE1 in regulating mitochondrial biogenesis and maintaining drug sensitivity in CRC, highlighting its potential as a therapeutic target.

The role of HUWE1 in CRC remains controversial, with evidence supporting both tumor-promoting and tumor-suppressing functions [37]. In this study, we showed that HUWE1 loss leads to increased cell proliferation through hyperactivation of Wnt/β-catenin signaling pathways. Although our study was only validated in in vitro models, it is consistent with previous reports showing that HUWE1 deletion accelerates CRC tumor formation [15, 38]. Additionally, we found that survival analysis using TCGA data and the Kaplan–Meier plotter revealed that low HUWE1 expression in CRC with deleterious APC mutations is associated with poor prognosis (Fig. 8C). Therefore, we suggest that HUWE1 loss in CRC will increase proliferation and EMT in vivo as well.

Conversely, other studies suggested that HUWE1 can promote tumorigenesis by mediating the ubiquitination of key oncogenic and tumor-suppressive proteins, such as c-Myc and p53 [29, 30]. HUWE1 plays a critical role in maintaining cellular homeostasis by regulating the stability and activity of various substrate proteins [12, 39]. Therefore, we propose that HUWE1 exhibits a dual role in CRC cell proliferation, with its function depending on the mutational status of its interacting substrates. Furthermore, the tumor-suppressive effect of HUWE1 may be context-dependent, occurring specifically under conditions where β-catenin is highly accumulated as a result of Wnt pathway hyperactivation, as observed in our study. Further research is needed to determine whether this regulatory function of HUWE1 is tissue-specific or confined to pathological states characterized by aberrant Wnt signaling.

In summary, we demonstrate that HUWE1 deficiency, in the context of β-catenin destruction complex dysfunction in CRC, enhances the Wnt/β-catenin signaling and mitochondrial biogenesis. Additionally, HUWE1 depletion leads to a deficiency in mitophagy, resulting in excessive accumulation of mitochondria and the overproduction of ATP, which activates ABC transporters. This leads to the induction of CSCs properties, EMT, increased cell proliferation, and chemoresistance (Fig. 8D). Additionally, when the β-catenin destruction complex functions normally, HUWE1 interacts with proteins such as Dvl and PPDPF. These findings uncover a novel mechanism by which HUWE1 regulates Wnt/β-catenin signaling, providing insights that may aid in identifying colorectal CSCs based on HUWE1 expression status.

## Materials and methods

Detailed materials and methods information is provided in the Supplementary information.

### Cell culture and establishment of HUWE1-deficient CRC cells

DLD1, HCT116, HT29, MCF7, HepG2, HeLa, and A549 cells were purchased from the Korean Cell Line Bank (KCLB, Seoul, Korea). RKO cells were kindly provided by Dr. HS Kim [40]. DLD1, HCT116, HT29, and A549 cells were cultured in RPMI1640 (Gibco, Grand Island, USA) supplemented with 10% FBS and 1% Penicillin/Streptomycin. MCF7, HepG2, HeLa, and RKO cells were cultured in DMEM (Gibco) supplemented with 10% FBS, and 1% Penicillin/Streptomycin. All cells were cultured in a humidified incubator with 5% CO_2_ at 37 °C. To establish the HUWE1-deficient CRC cell model, we generated stable HUWE1 knockdown CRC cells using lentiviral vectors containing either shHUWE1 or scrambled shRNA as a control, following the standard protocol previously used for viral delivery [41]. Subsequently, after appropriate selection steps, a Western blot analysis was performed on stable cell lines to test for HUWE1 expression.

### Statistical analysis

All the experiments with cells were repeated at least three times. The results are presented as the mean ± SEM. The *p*-value was calculated using Student’s *t* test, and the *p*-values lower than 0.05 were considered statistically significant. Pearson correlation analysis was used to assess statistical significance for differences between mRNA levels in the TCGA data set. Statistical analysis was performed using GraphPad software version 6.00 (GraphPad Prism Software, San Diego, USA).

## Supplementary information

Supplementary Information

Original Western blot

## Data Availability

All data supporting the findings of this study can be freely accessed by any researcher for non-commercial purposes upon reasonable request.
