# Supplementary material for: HUWE1 loss promotes stemness and drug resistance in CRC with dysregulated β-catenin destruction complex
Source: Cell Death Discov. 2025 Oct 6;11:424. doi: 10.1038/s41420-025-02731-2 (PMC12500962; doi:10.1038/s41420-025-02731-2)
Supplement: Supplementary file 1 — Supplementary Information [file 41420_2025_2731_MOESM1_ESM.docx]

**HUWE1 loss promotes stemness and drug resistance in CRC with dysregulated β-catenin destruction complex**

Chanhaeng Lee^1,2,3^, Sang-Hee Park^2^, Inn-Oc Han^4,5^, Sungjoo Kim Yoon^1,2*^

^1^Department of Medical Sciences, ^2^Department of Medical Life Sciences, College of Medicine, The Catholic University of Korea, 222 Banpo-daero, Seocho-gu, Seoul, 065-691. ^3^BK21 Center for Precision Medicine & Smart Engineering, ^4^Department of Biomedical Science, Program in Biomedical Science and Engineering, ^5^Department of Physiology and Biophysics, College of Medicine, Inha University, 100 Inha-ro, Michuhol-gu, Incheon, 22212

Republic of Korea.

^*^**Corresponding Author’s Information:**

Tel: +82-2-3147-8398; E-mail address: sjkyoon@catholic.ac.kr

**Supplementary Information**

**Supplementary Figures**

| **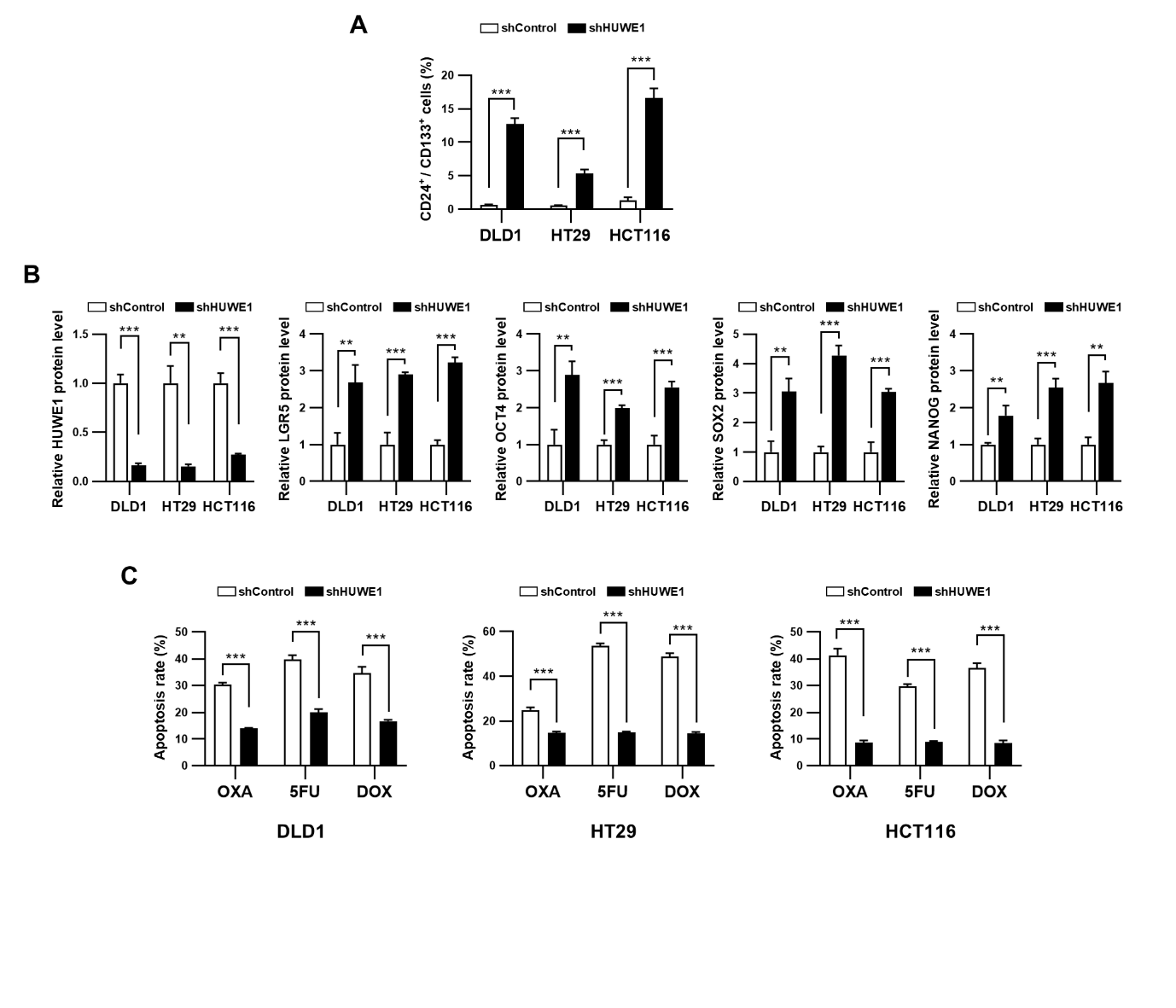** |
| --- |
| **Supplementary Figure 1.** **(A)** Comparison of the ratios of CD24^+^/CD133^+^ cells quantified by FACS analysis in DLD1, HT29, and HCT116 cells with or without HUWE1 depletion. **(B)** Quantification of the indicated proteins shown in Figure 1D. **(C)** Comparison of the apoptosis rates in DLD1, HT29, and HCT116 cells with or without HUWE1 depletion following treatment with oxaliplatin, 5FU, and doxorubicin. Data are presented as mean ± SEM. **P* < 0.05, ***P* < 0.01, ****P* < 0.001. |

| **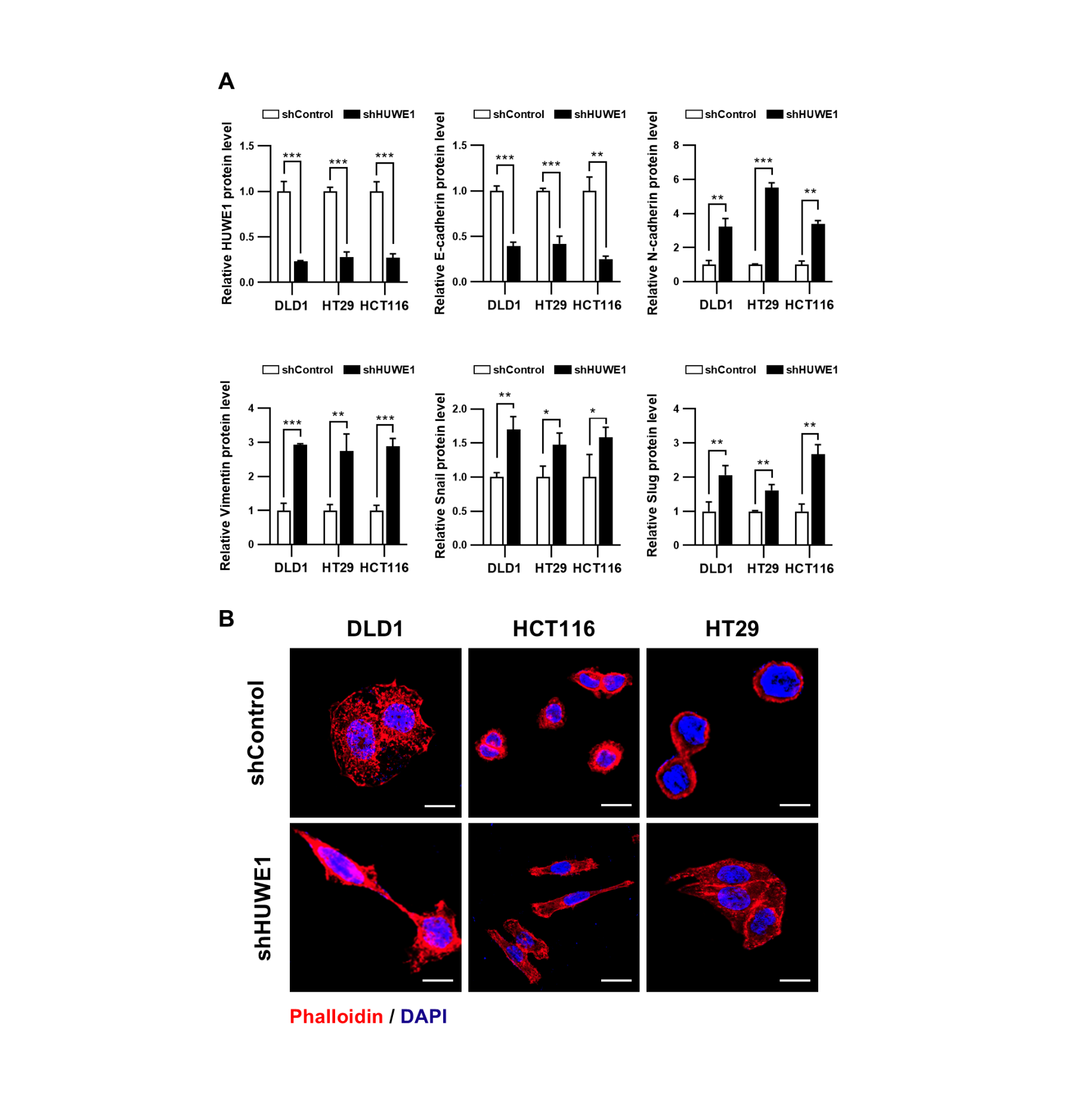** |
| --- |
| **Supplementary Figure 2.** **(A)** Quantification of the indicated proteins shown in Figure 2F. **(B)** Cell morphology analysis using Phalloidin-iFluor 647 staining in DLD1, HT29, and HCT116 cells, with or without HUWE1 depletion. Scale bar, 20 µm. Data are presented as mean ± SEM. **P* < 0.05, ***P* < 0.01, ****P* < 0.001. |

**
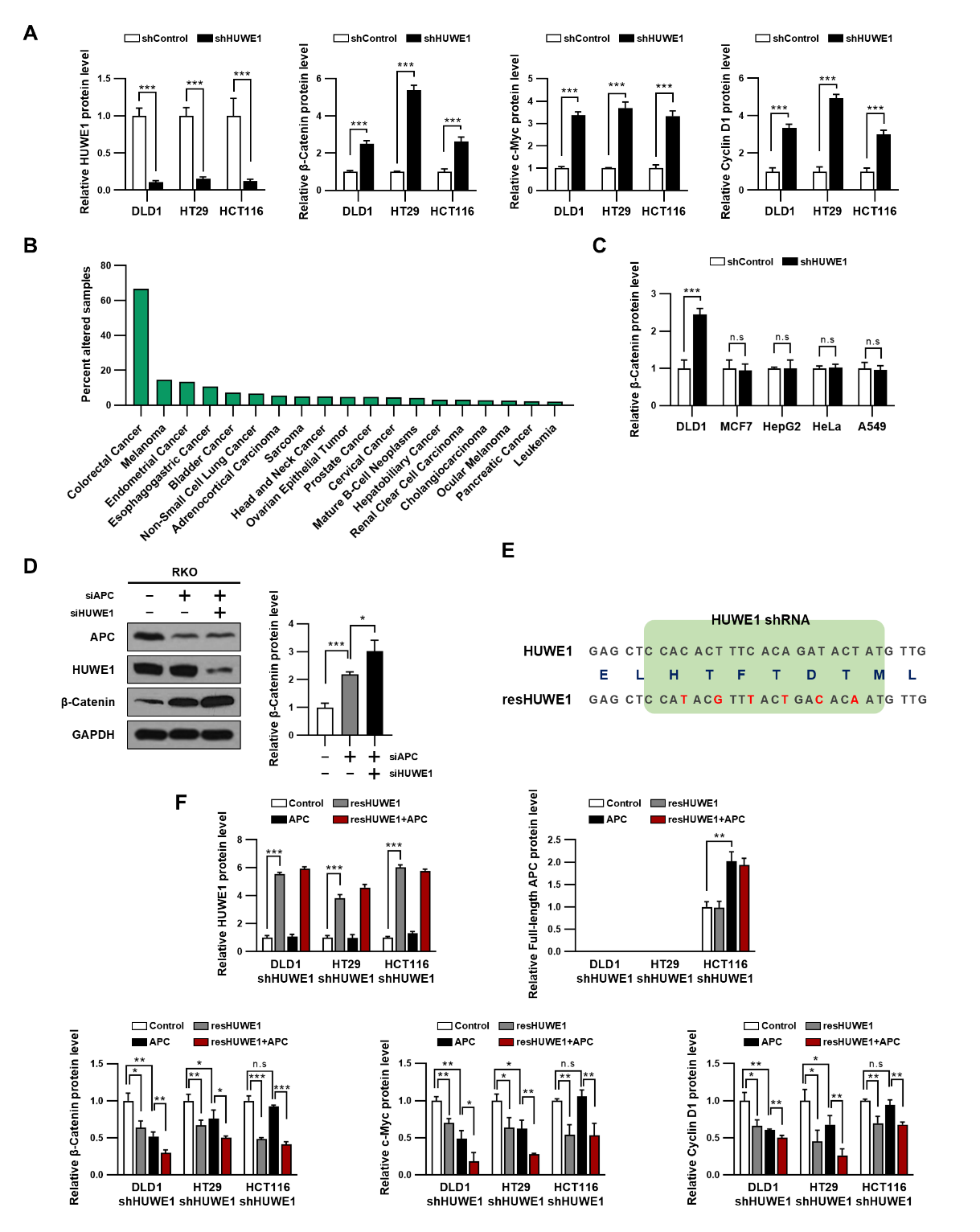
Supplementary Figure 3. (A)** Quantification of the indicated proteins shown in Figure 3B. **(B)** cBioPortal OncoPrints of *APC* mutation rates across various human cancers. **(C)** Quantification of the β-Catenin protein shown in Figure 3D. **(D)** Comparison of β-Catenin protein levels in RKO cells with APC inhibition alone or with simultaneous inhibition of both APC and HUWE1. **(E)** Nucleotide sequence of resHUWE1 preventing knockdown by HUWE1 shRNA. **(F)** Quantification of the indicated proteins shown in Figure 3F. Data are presented as mean ± SEM. **P* < 0.05, ***P* < 0.01, ****P* < 0.001.


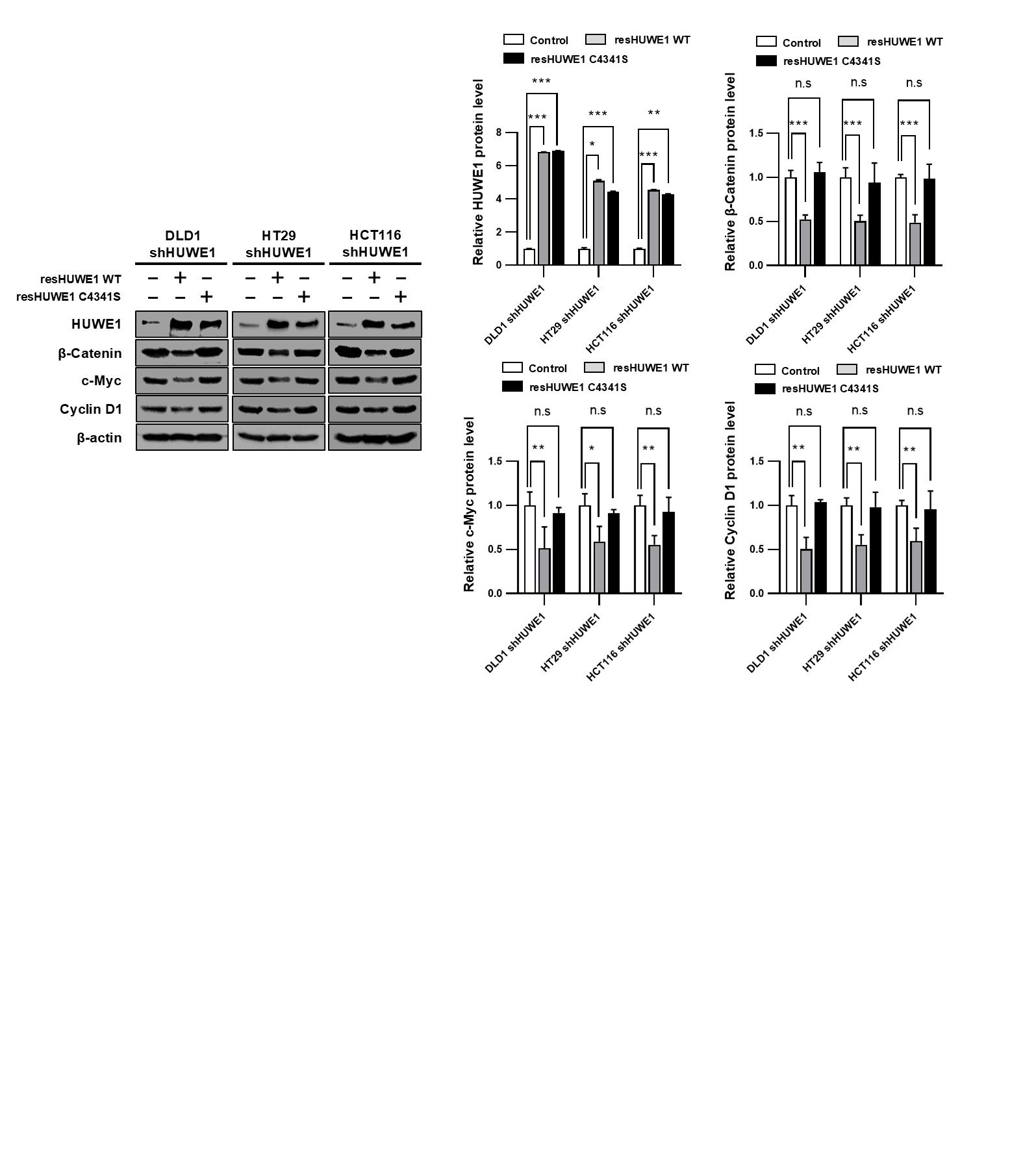


**Supplementary Figure 4.** Western blot analysis detected the expression of β-catenin, c-Myc, and Cyclin D1 in HUWE1-deficient CRC cells overexpressing either resHUWE1 WT or the catalytically inactive mutant resHUWE1 C4341S. Data are presented as mean ± SEM. **P* < 0.05, ***P* < 0.01, ****P* < 0.001.

| **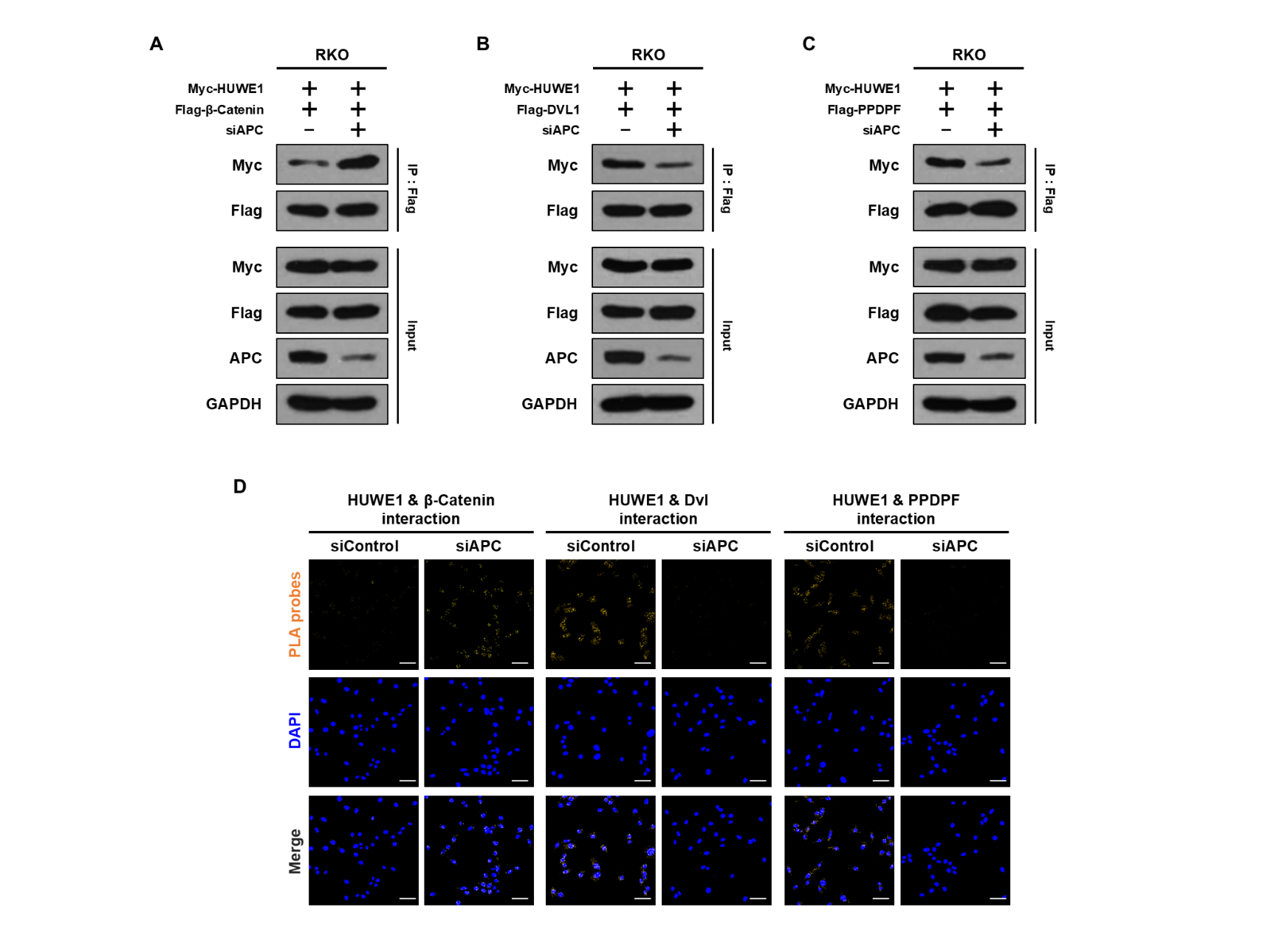Supplementary Figure 5.** **(A–C)** Comparison of the interaction between HUWE1 and β-catenin (A), DVL1 (B), or PPDPF (C) in RKO cells transfected with control siRNA or *APC* siRNA using Co-immunoprecipitation assay. **(D)** PLA signals were observed in RKO cells transfected with control siRNA or *APC* siRNA by confocal microscopy. Scale bar, 50 µm. |
| --- |

| **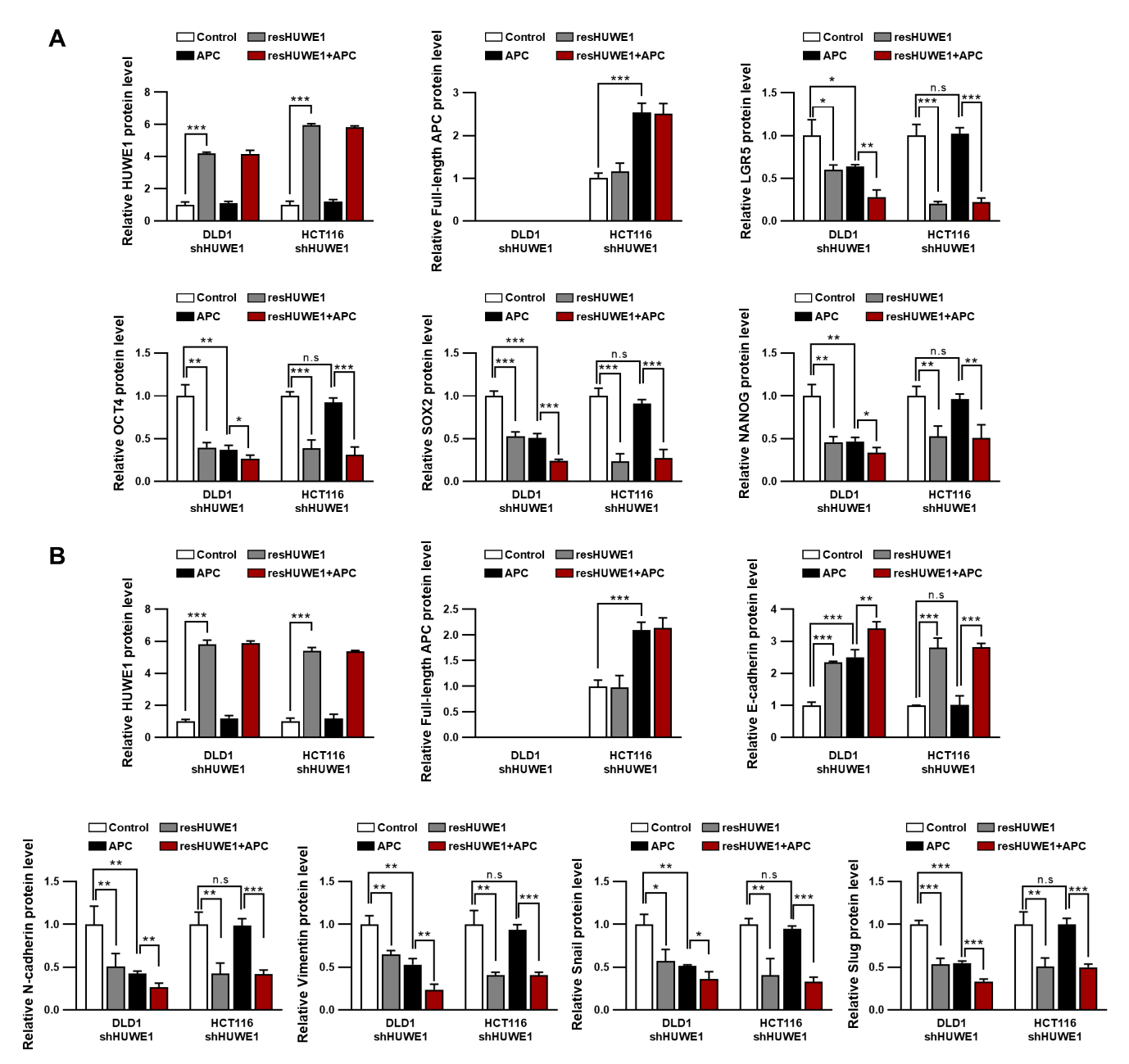** |
| --- |
| **Supplementary Figure 6.** **(A, B)** Quantification of the indicated proteins shown in Figure 5G (A) and Figure 5H (B). Data are presented as mean ± SEM. **P* < 0.05, ***P* < 0.01, ****P* < 0.001. |


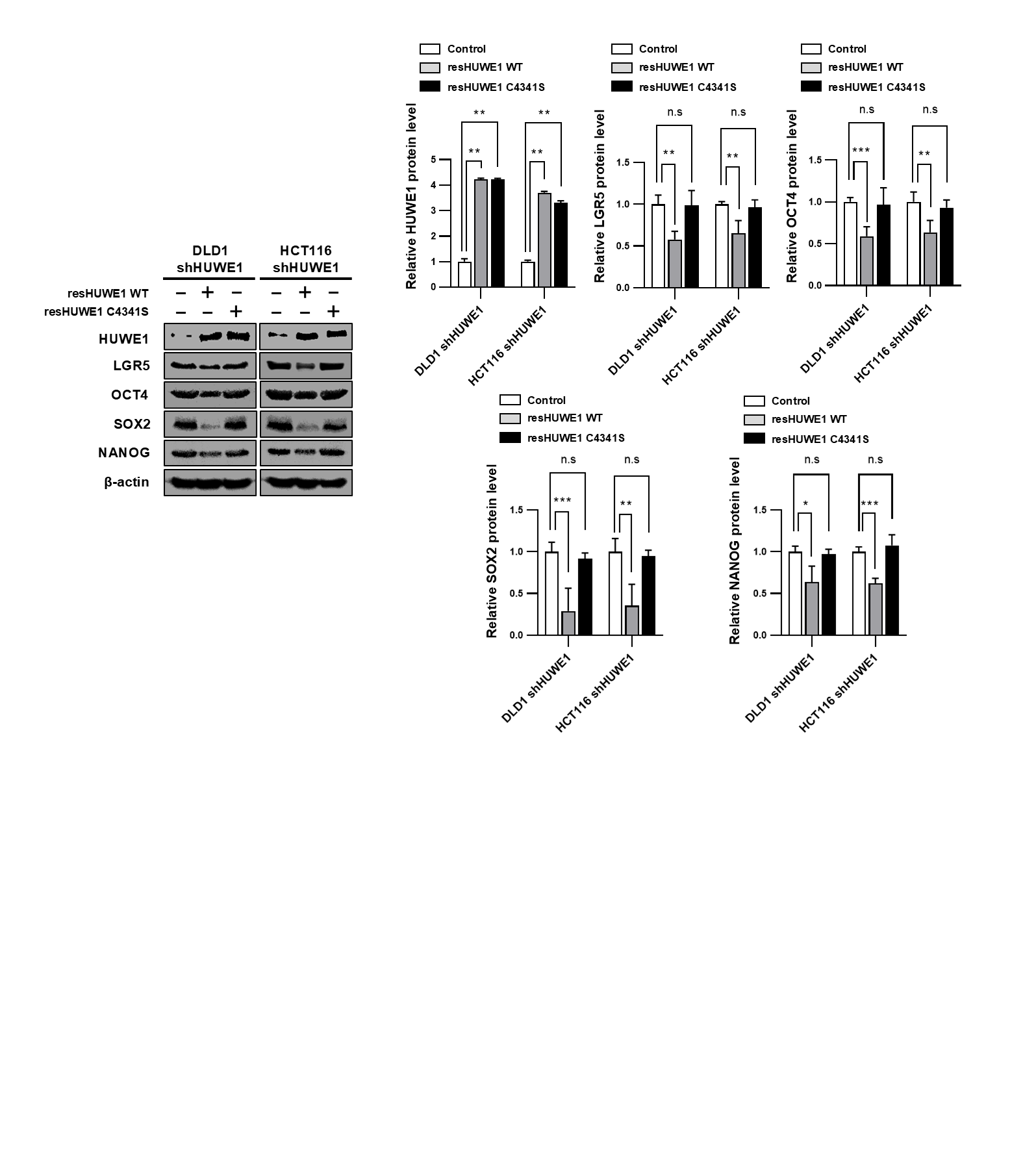
**Supplementary Figure 7.** Western blot analysis detected the expression of LGR5, OCT4, SOX2, and NANOG in HUWE1-deficient DLD1 and HCT116 cells overexpressing either resHUWE1 WT or the catalytically inactive mutant resHUWE1 C4341S. Data are presented as mean ± SEM. **P* < 0.05, ***P* < 0.01, ****P* < 0.001.

| **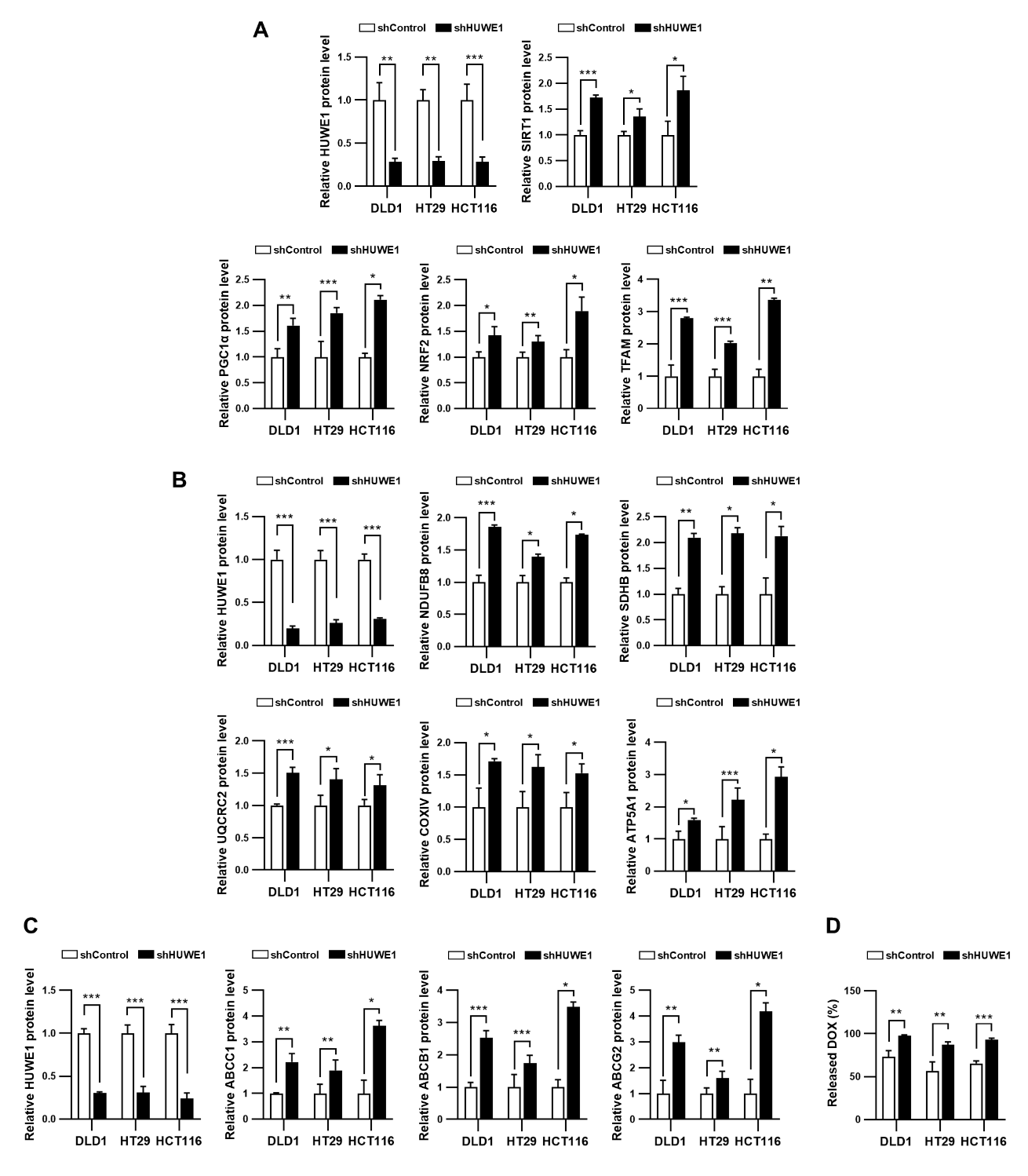** |
| --- |
| **Supplementary Figure 8. (A-C)** Quantification of the indicated proteins shown in Figure 6A (A), Figure 6B (B), and Figure 6H (C). **(D)** Fluorescence levels were measured after treatment with 10 µM doxorubicin for 4 hrs in DLD1, HT29, and HCT116 cells with or without HUWE1 depletion. After 24 hrs, fluorescence was measured again, and the amount of doxorubicin released was calculated as a percentage. Data are presented as mean ± SEM. **P* < 0.05, ***P* < 0.01, ****P* < 0.001.  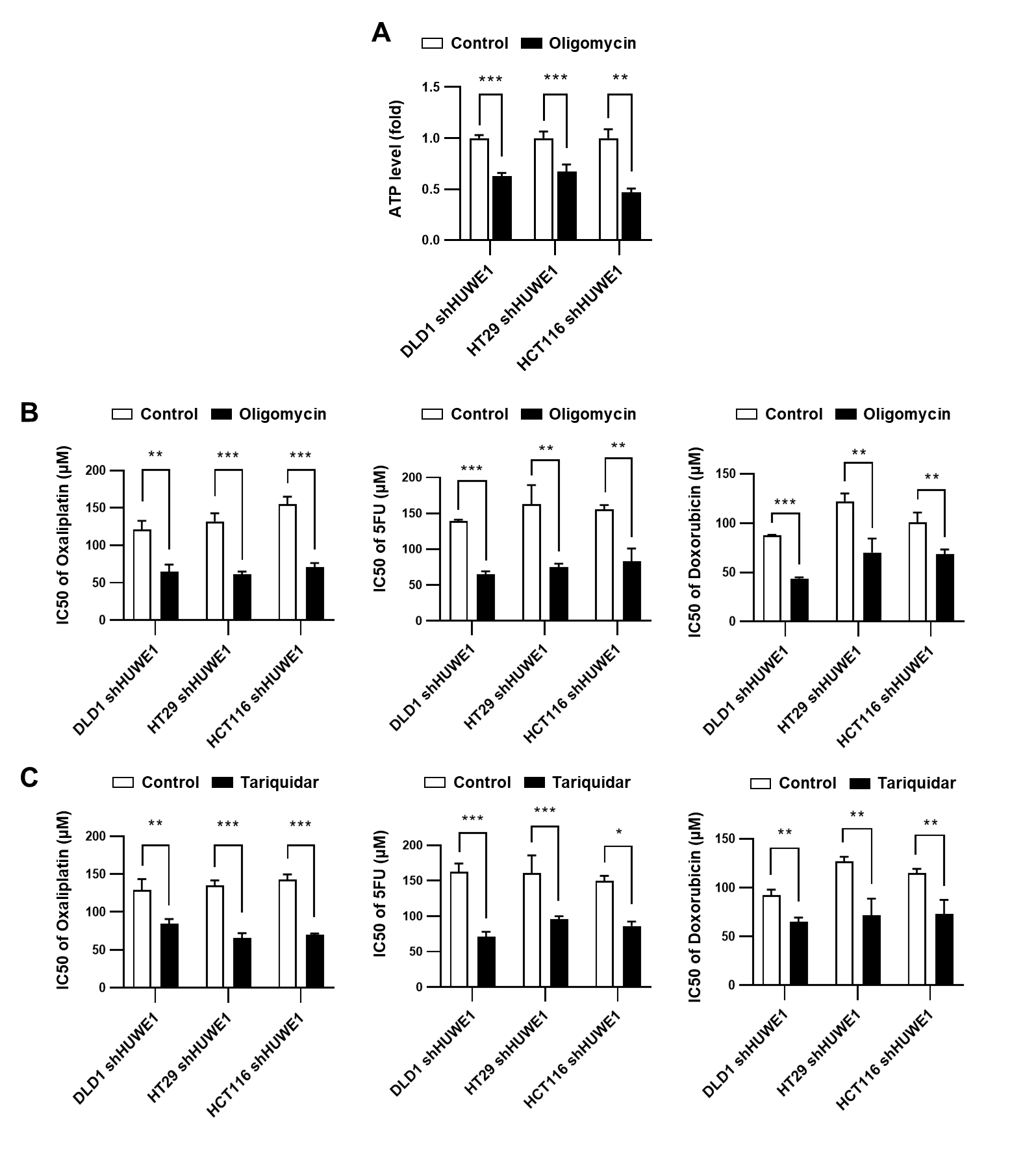 |

**Supplementary Figure 9. (A)** Measurement of ATP level after oligomycin treatment in HUWE1-deficient CRC cells. **(B)** The IC50 of oxaliplatin, 5FU, and doxorubicin after oligomycin treatment in HUWE1-deficient cells was determined using the CCK-8 assay. **(C)** The IC50 of oxaliplatin, 5FU, and doxorubicin after tariquidar treatment in HUWE1-deficient cells was determined using the CCK-8 assay. Data are presented as mean ± SEM. **P* < 0.05, ***P* < 0.01, ****P* < 0.001.

| 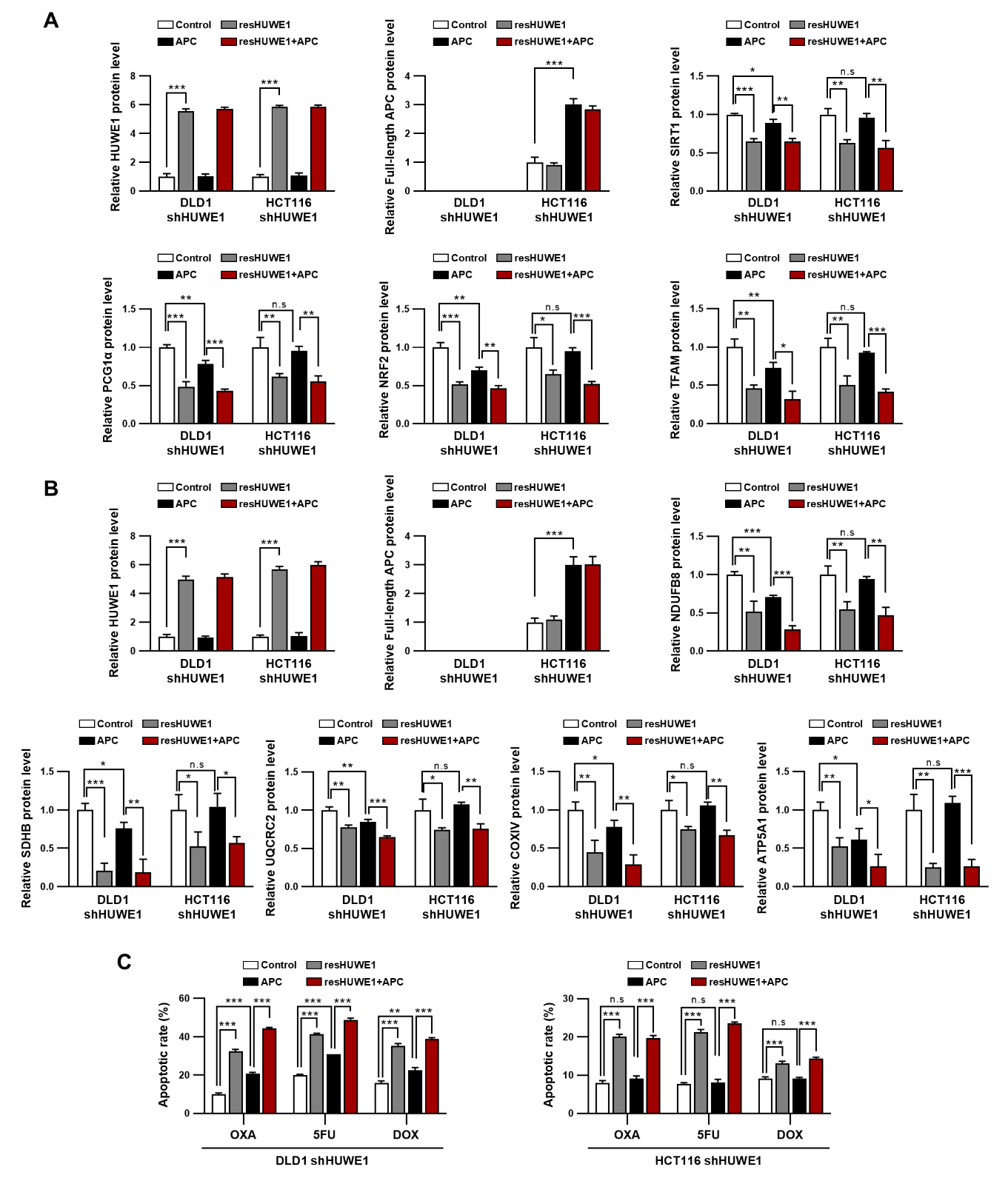 |
| --- |
| **Supplementary Figure 10. (A, B)** Quantification of the indicated proteins shown in Figure 7A (A) and Figure 7B (B). **(C)** Comparison of apoptotic cell rates shown in Figure 7H. Data are presented as mean ± SEM. **P* < 0.05, ***P* < 0.01, ****P* < 0.001. |

**Supplementary materials and methods**

**Construction of expression plasmids and transfections**

For generating HUWE1 knockdown CRC cell lines, shRNA against HUWE1 was kindly provided by Dr. HS Cheong [1]. The Myc-tag HUWE1 expression plasmid was used in a previous study [2]. To construct the resHUWE1 expression plasmid, the *HUWE1* fragment was generated by PCR using the pCMV6-HUWE1 expression plasmid from a previous study as a template [2]. Additionally, we generated a *resHUWE1* fragment with six-point mutations within the shRNA target DNA sequence, then replaced it in the *HUWE1* fragment in the correct orientation using the SacI restriction sites. The completed fragment was inserted into the pCMV6-HUWE1 expression plasmid using XbaI and MluI restriction sites, generating the resHUWE1 plasmid. To construct the resHUWE1 C4341S expression plasmid, we utilized the HUWE1 C4341S plasmid used in a previous study. [2]. We obtained fragments from the resHUWE1 plasmid using XbaI and MluI restriction sites, which were then inserted into the pCMV6-HUWE1 C4314S expression plasmid to generate the resHUWE1 C4341S plasmid. *Flag-β-Catenin* and *Flag-PPDPF* cDNA were generated by PCR using isoform 1 of *β-Catenin* (GenBank: NM_001904.4) and *PPDPF* (GenBank: NM_024299) as a template. *Flag-DVL1* cDNA was generated by PCR using the pDONR223_DVL1_WT plasmid (Plasmid #82110) as a template. Each resulting Flag tag fragment was inserted into the pCMV6-Entry-Myc-DDK mammalian expression plasmid (Origene, #PS100001) using EcoRI and EcoRV restriction sites. The full-length *APC* cDNA was generated by PCR using isoform a of *APC* (GenBank: NM_001127511.3). The full-length *APC* cDNA was inserted into the pCMV6-Entry-Myc-DDK mammalian expression plasmid using SalI and NotI restriction sites, which removed Myc and Flag tags.

The constructed plasmids were utilized for transfection experiments. *HUWE1* siRNA was used in a previous study [2]. *APC* siRNA (sc-29702) was purchased from Santa cruz (Texas, USA). All cells were transfected with siRNA at a final concentration of 100 nM. All transfection experiments were performed using Lipofectamine 2000 (Invitrogen, Carlsbad, USA) according to the manufacturer’s protocol. Cells were harvested at 48 or 72 hrs post-transfection and used for protein extraction. The sequences of the oligonucleotides used for cloning are shown in Supplementary Table S1.

**Tumor spheroid formation assay**

Cells were cultured to 80% confluence and then dissociated into a single-cell suspension using trypsin. The single-cell suspension was then diluted in fresh 3D Tumorsphere Medium XF (Sigma-Aldrich, St. Louis, USA) to a concentration of 10 viable cells per mL. Then, 100 μL of the single-cell suspension was distributed into each well of the spheroid microplates (Corning, New York, USA). The plates were cultured at 37 °C for 10 days, and the spheroid diameters were quantified using an optical microscope.

**Colony formation assay**

Cells were suspended and plated in a 60 mm culture dish at a density of 10^3^ cells. After 2 weeks of cultivation, the cells were fixed with 4% paraformaldehyde for 30 min and stained with 0.5% crystal violet. The relative number of colonies was then counted.

**FACS analysis**

Cells were harvested and washed with FACS buffer (DPBS containing 0.2% BSA), and then incubated with antibodies against CD133-FITC (BD Biosciences, 567029) and CD24-PE (BD Biosciences, 555428) for 30 min on ice. After incubation, the samples were washed three times with cold FACS buffer, and analysis was performed using a BD FACS Canto II flow cytometer (BD Biosciences, Franklin Lakes, USA).

For apoptosis analysis, an apoptosis detection kit (BD Biosciences, 556547) was used. Cells were incubated with Annexin V-FITC and PI for 20 min, then sorted using a BD FACS system according to the manufacturer's instructions. Data were analyzed using BD FACSAria™ software (BD Biosciences) and FlowJo software for further analysis. The apoptotic rate was calculated as the sum of early apoptotic cells (Annexin V+/PI-) and late apoptotic cells (Annexin V+/PI+).

**Western blot analysis**

Total proteins were extracted from cells using RIPA lysis buffer with a protease inhibitor cocktail (GenDEPOT, Barker, USA) by lysis on ice for 30 min. The lysates were centrifuged, and protein concentration was measured using the Bradford assay (Bio-rad, Hercules, USA). Equal amounts of denatured proteins were separated by SDS-PAGE, transferred to a nitrocellulose membrane, and blocked with 5% non-fat milk. After overnight incubation with a primary antibody, the membrane was incubated with an HRP-conjugated secondary antibody and visualized using the ECL system (DonginBiotech, Seoul, Korea). Band intensity was quantified using ImageJ software. The primary antibodies used are listed in Supplementary Table S2.

**Cell viability detection**

To assess cell viability, a CCK-8 assay (DonginBiotech) was performed. Cells were suspended and plated in a 96-well plate at a density of 10^4^ cells per well with various concentrations of oxaliplatin, 5FU, or doxorubicin (Sigma-Aldrich). To evaluate the effects of mitochondrial ATP production and ABC transporter protein activity on drug sensitivity, the cells were pretreated with oligomycin 10 µM and tariquidar 5 µM (MedChemExpress, New Jersey, USA) for 6 hrs. Oligomycin and tariquidar were not removed, and additional drugs were added. After 48 hrs, the cells were incubated with CCK-8 agent in culture medium for 30 min at 37 °C. Absorbance at 450 nm was measured using a microplate reader (Bio-Tek, Winooski, USA). IC50 values were calculated by determining the linear equation at 50% cell viability and setting the Y value to 0.5.

**Cell proliferation assay**

To measure cell proliferation, a CCK-8 assay (DonginBiotech) was performed. Cells were suspended and seeded in a 96-well plate at a density of 10^4^ cells per well. Every 24 hrs for 5 days, the cells were incubated with CCK-8 agent in culture medium for 30 min at 37 °C. Absorbance at 450 nm was measured using a microplate reader (Bio-Tek). EdU cell proliferation was performed using VF 488 Click-iT EdU Universal Cell Proliferation Detection Kit (MedChemExpress). The cells were cultured on a coverslip and incubated with 10 µM EdU for 4 hrs. After fixation, experiments were performed according to the manufacturer’s protocol, and cells were observed using a confocal microscope (LSM800W/Airyscan, Carl Zeiss, Oberkochen, Germany).

**Wound-healing assay**

To assess cell migration, a wound healing assay was performed. After reaching 90-100% confluence, the cells were treated with 5 µg/ml mitomycin C. The cell monolayer was scratched in a straight line using a 200 µl sterile yellow tip, followed by careful washing to remove detached cells. The cells were subsequently incubated at 37 °C. Images of the scratch wounds for each sample were taken at 0 and 24 hours using a digital camera mounted on a microscope. The wound closure rate was measured using ImageJ software.

**Cell invasion assay**

The invasion assay was carried out with Matrixgel-coated transwell chambers (Corning). Cells (5 x 10^4^ cells) were suspended in 100 µl of serum-free medium in the upper chamber, which was then placed over the lower chamber filled with 600 µl of culture medium supplemented with 10% FBS. After 24 hours of incubation, the invaded cells were fixed with 4% paraformaldehyde for 30 min and stained with 0.1% crystal violet. The invaded cells were counted under a microscope in three fields per well.

**Immunofluorescence**

Cells were fixed in 4% paraformaldehyde at room temperature for 30 min. The fixed cells were permeabilized with 0.1% Triton X-100 for 10 min, blocked in 2% BSA dissolved in DPBS, and then incubated overnight at 4 °C with the primary antibody. After washing with the blocking buffer, the cells were incubated with an Alexa Fluor 594-conjugated secondary antibody for 1 hr. Nuclei were stained with DAPI and observed using a confocal microscope (LSM800W/Airyscan, Carl Zeiss).

To observe morphological changes in the cells, the fixed cells were permeabilized and incubated with Phalloidin-iFluor 647 Reagent (Abcam, ab176759) for 1 hr at room temperature. Nuclei were stained with DAPI and observed using a confocal microscope (LSM800W/Airyscan, Carl Zeiss).

**Immunoprecipitation**

To verify protein-protein interactions, cells were lysed in 1% NP-40 buffer (50 mM Tris-HCl (pH 7.4), 150 mM NaCl, 1 mM EDTA, 1% NP-40, and Protease inhibitor cocktail) without SDS to maintain protein interactions for the co-immunoprecipitation experiment. For ubiquitination analysis, cells were treated with 25 µM MG132 for 6 hours, harvested, and lysed in RIPA buffer. The lysate was centrifuged at 12,000 × *g* for 15 min at 4 °C. The supernatant was incubated overnight at 4 °C with anti-FLAG M2 agarose beads (Sigma-Aldrich). After centrifugation, the pellet was washed with 1% NP-40 buffer and then eluted with SDS sample buffer. The eluted proteins were separated by SDS-PAGE, transferred to a nitrocellulose membrane, and analyzed by Western blotting.

**Proximity ligation assay**

The PLA experiment was performed using the Duolink® in situ orange starter kit Mouse/Rabbit (Sigma-Aldrich) according to the manufacturer’s instructions. The cells were incubated with primary Rabbit anti-HUWE1 antibody (Bethyl, A300-486A) and Mouse anti-β-Catenin antibody (Santa cruz, sc-7963), primary Rabbit anti-HUWE1 antibody (Bethyl, A300-486A) and Mouse anti-Dvl antibody (Santa cruz, sc-166303), or primary Rabbit anti-PPDPF antibody (Cusabio, CSB-PA003681GA01HU) and Mouse anti-HUWE1 antibody (Cell signaling, 5695), respectively, overnight at 4 °C. After washing the cells, Duolink® in situ PLA® probe Rabbit PLUS probe and Duolink® in situ PLA® probe Mouse MINUS were incubated, followed by ligation and amplification. PLA probe (Orange) was observed using a confocal microscope (LSM800W/Airyscan, Carl Zeiss) at 576 nm.

**ETC Enzyme activity assay**

Complex I activity was measured using the Complex I Enzyme Activity Microplate Assay Kit (Abcam, ab109721), according to the manufacturer’s instructions. Complex I-specific antibody is pre-coated on the microplate well, with the target immobilized. Complex I activity was determined by oxidizing NADH to NAD^+^, simultaneously reducing the provided dye, and measuring the increase in absorbance at 450 nm. Complex I activity was measured using 300 µg of total protein per sample, resuspended in 200 µL of assay buffer.

Complex IV activity was measured using the Complex IV Human Enzyme Activity Microplate Assay Kit (Abcam, ab109909), according to the manufacturer’s instructions. Complex IV-specific antibody is pre-coated on the microplate well with the target immobilized. Complex IV activity was determined by oxidation of reduced cytochrome c, measured as a decrease in absorbance at 550 nm. Complex IV activity was measured using 100 μg of total protein per sample, resuspended in 200 µL of assay buffer.

ATP synthase (Complex V) activity assay was measured using the ATP synthase Enzyme Activity Microplate Assay kit (Abcam, ab109714), according to the manufacturer’s instructions. Complex V-specific antibody is pre-coated on the microplate well with the target immobilized. Complex V activity was determined by oxidizing NADH to NAD^+^ and measuring the decrease in absorbance at 340 nm. Complex V activity was measured using 100 µg of total protein per sample, resuspended in 200 µL of assay buffer. Enzyme activity was calculated from the change in absorbance using the extinction coefficient of each dye.

**Measurement of ATP amount**

The total ATP content was measured using an ATP analysis kit (Invitrogen) according to the manufacturer's instructions. Briefly, the cells were washed with DPBS and then boiled in pure H_2_O for 10 min. The amount of ATP was measured using a luminometer (Spectra Max, Molecular Devices, San Jose, USA).

**Measurement of intracellular accumulation of doxorubicin**

Cells were cultured with 10 µM doxorubicin on coverslips for 24 hrs. On the following day, the cells were washed with DPBS and fixed. Nuclei were stained with DAPI, and the red fluorescence of doxorubicin was observed using a confocal microscope. (LSM800W/Airyscan, Carl Zeiss). To measure the amount of doxorubicin released, cells were plated in 96-well plates at a density of 10^4^ cells per well and treated with 10 µM of doxorubicin. Doxorubicin release was calculated as a percentage by measuring the initial fluorescence after 4 hrs and subtracting the fluorescence measured after 24 hrs. The fluorescence of doxorubicin was measured using a luminometer (Spectra Max, Molecular Devices).

**Clinical Data Profiling**

The correlation analysis between *HUWE1* and mitochondrial protein expression, as well as the analysis of *HUWE1* expression based on the presence or absence of APC mutations, was performed using publicly available TCGA (The Cancer Genome Atlas) datasets queried via cBioPortal (<https://www.cbioportal.org/>). The survival curves were generated using the Kaplan–Meier method via Xena Browser (<https://xenabrowser.net/heatmap/>) from the University of California, Santa Cruz (UCSC). The differences in *HUWE1* expression in survival outcomes were stratified into high and low groups based on quartiles and analyzed using the log-rank test.

**Supplementary Tables**

**Supplementary Table S1.**

| Primer name | Sequence (5’🡪3’) | |
| --- | --- | --- |
| Primers for missense plasmids construction | | |
| *HUWE1* fragment | forward primer | CCTGTTTCTGAGGGATTGGA |
|  | reverse primer | GGCTGAGGTTGGAAGGAG |
| *resHUWE1* fragment | forward primer | GAGCTCCATACGTTTACTGACACAATGTT |
|  | reverse primer | ATTGAAGCCTGAGCTCTGGGTC |
| *Flag-β-Catenin* | forward primer | AGGGACCATGGCTACTCAAGCTGATTTGATGGAG |
|  | reverse primer | GATATCCAGGTCAGTATCAAACCAGGCC |
| *Flag-PPDPF* | forward primer | AAGCGAAAGCATGGCGGCCAT |
|  | reverse primer | GATATCGGACGGGGGCCCA |
| *Flag-DVL1* | forward primer | CCATGGCGGAGACCAAGATTAT |
|  | reverse primer | GATATCCATGATGTCCACGAAGAACT |
| *APC* | forward primer | GCCACCATGTACGCCTCCCT |
|  | reverse primer | CCTCTCTTTTAAACAGATGTCACAAGG |

**Supplementary Table S2.**

**List of Primary antibodies in this study**

| **Antibody** | **Catalog Number** | **Company** |
| --- | --- | --- |
| HUWE1 | A300-486A | Bethyl |
| LGR5 | CSB-PA622827 | Cusabio |
| OCT4 | sc-9081 | Santa cruz |
| SOX2 | CSB-PA16539A0Rb | Cusabio |
| NANOG | CSB-PA888008LA01HU | Cusabio |
| E-cadherin | CSB-PA002243 | Cusabio |
| N-cadherin | CSB-PA003379 | Cusabio |
| Vimentin | A301-620A | Bethyl |
| Snail | CSB-PA004123 | Cusabio |
| Slug | CSB-PA105844 | Cusabio |
| β-Catenin | CSB-PA00174A0Rb | Cusabio |
| c-Myc | sc-40 | Santa cruz |
| Cyclin D1 | CSB-PA001875 | Cusabio |
| APC | 2504 | Cell signaling |
| SIRT1 | CSB-PA004095 | Cusabio |
| PGC1α | A12348 | ABclonal |
| NRF2 | CSB-PA003481 | Cusabio |
| TFAM | CSB-PA050245 | Cusabio |
| NDUFB8 | CSB-PA015655LA01HU | Cusabio |
| SDHB | CSB-PA005802 | Cusabio |
| UQCRC2 | CSB-PA025668ESR1HU | Cusabio |
| COXIV | A6564 | ABclonal |
| ATP5A1 | A304-940T | Bethyl |
| ABCC1 | CSB-PA997657 | Cusabio |
| ABCB1 | CSB-MA00104640m | Cusabio |
| ABCG2 | CSB-PA000788 | Cusabio |
| Flag Probe | 14793S | Cell signaling |
| Myc Probe | 2276S | Cell signaling |
| HA Probe | sc-7392 | Santa cruz |
| GAPDH | sc-166574 | Santa cruz |
| β-actin | sc-47778 | Santa cruz |

**Supplementary References**

1. Lee J, Kim J, Shin J, Kang Y, Choi J, Cheong H. ATG101 Degradation by HUWE1-Mediated Ubiquitination Impairs Autophagy and Reduces Survival in Cancer Cells. Int J Mol Sci. 2021;22:9182.
2. Lee C, Park SH, Yoon SK. The E3 ligase HUWE1 increases the sensitivity of CRC to oxaliplatin through TOMM20 degradation. Oncogene. 2024;43:636-649.
