# Supplementary material for: HUWE1 loss promotes stemness and drug resistance in CRC with dysregulated β-catenin destruction complex
Source: Cell Death Discov. 2025 Oct 6;11:424. doi: 10.1038/s41420-025-02731-2 (PMC12500962; doi:10.1038/s41420-025-02731-2)

Figure 1D

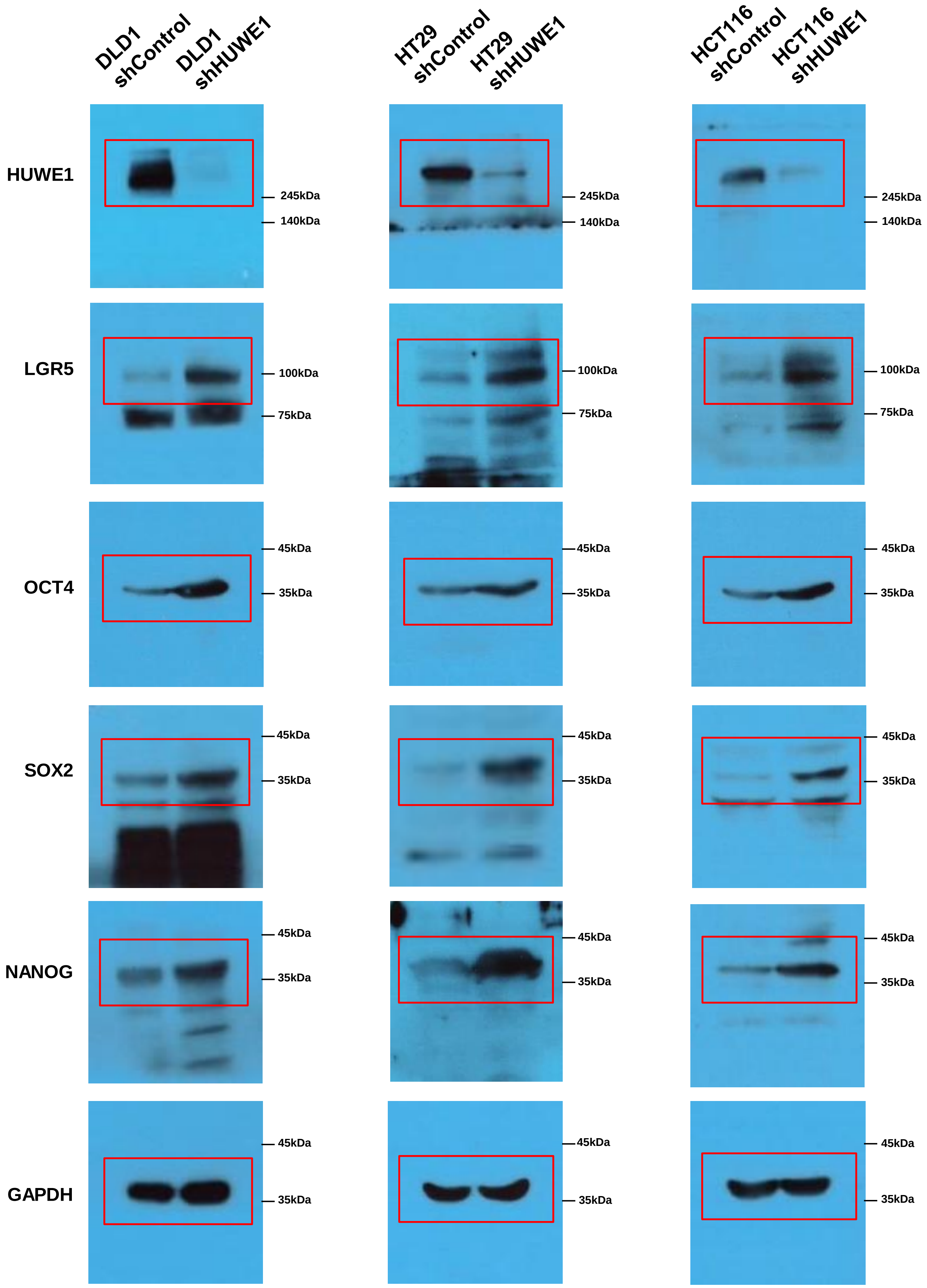
DLD1 shControl
DLD1 shHUWE1
HT29 shControl
HT29 shHUWE1
HCT116 shControl
HCT116 shHUWE1
HUWE1
LGR5
OCT4
SOX2
NANOG
GAPDH
245kDa
140kDa
100kDa
75kDa
45kDa
35kDa

Figure 2F

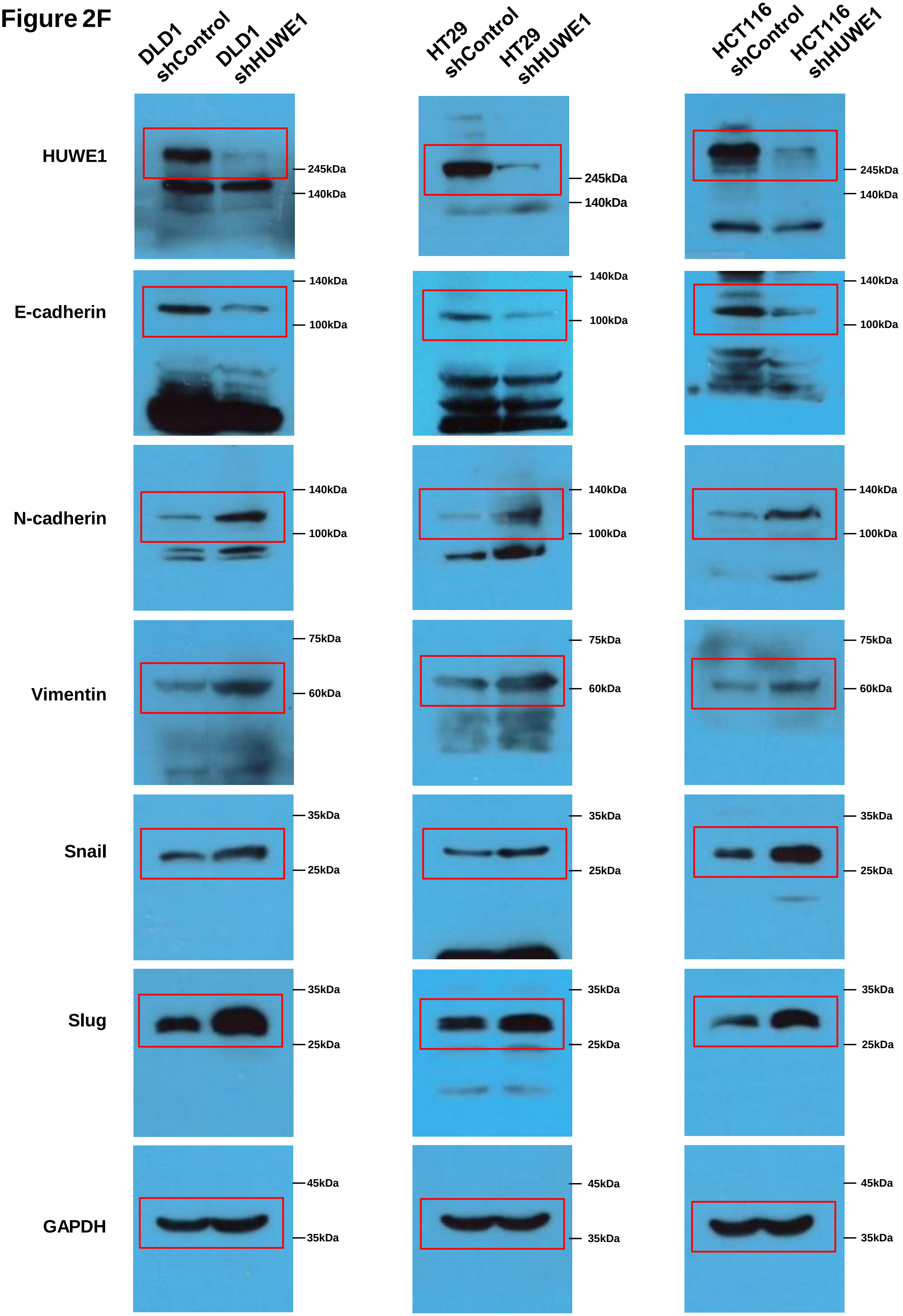
DLD1 shControl
DLD1 shHUWE1
HT29 shControl
HT29 shHUWE1
HCT116 shControl
HCT116 shHUWE1
HUWE1
E-cadherin
N-cadherin
Vimentin
Snail
Slug
GAPDH
245kDa
140kDa
100kDa
75kDa
60kDa
35kDa
25kDa
45kDa

Figure 3B

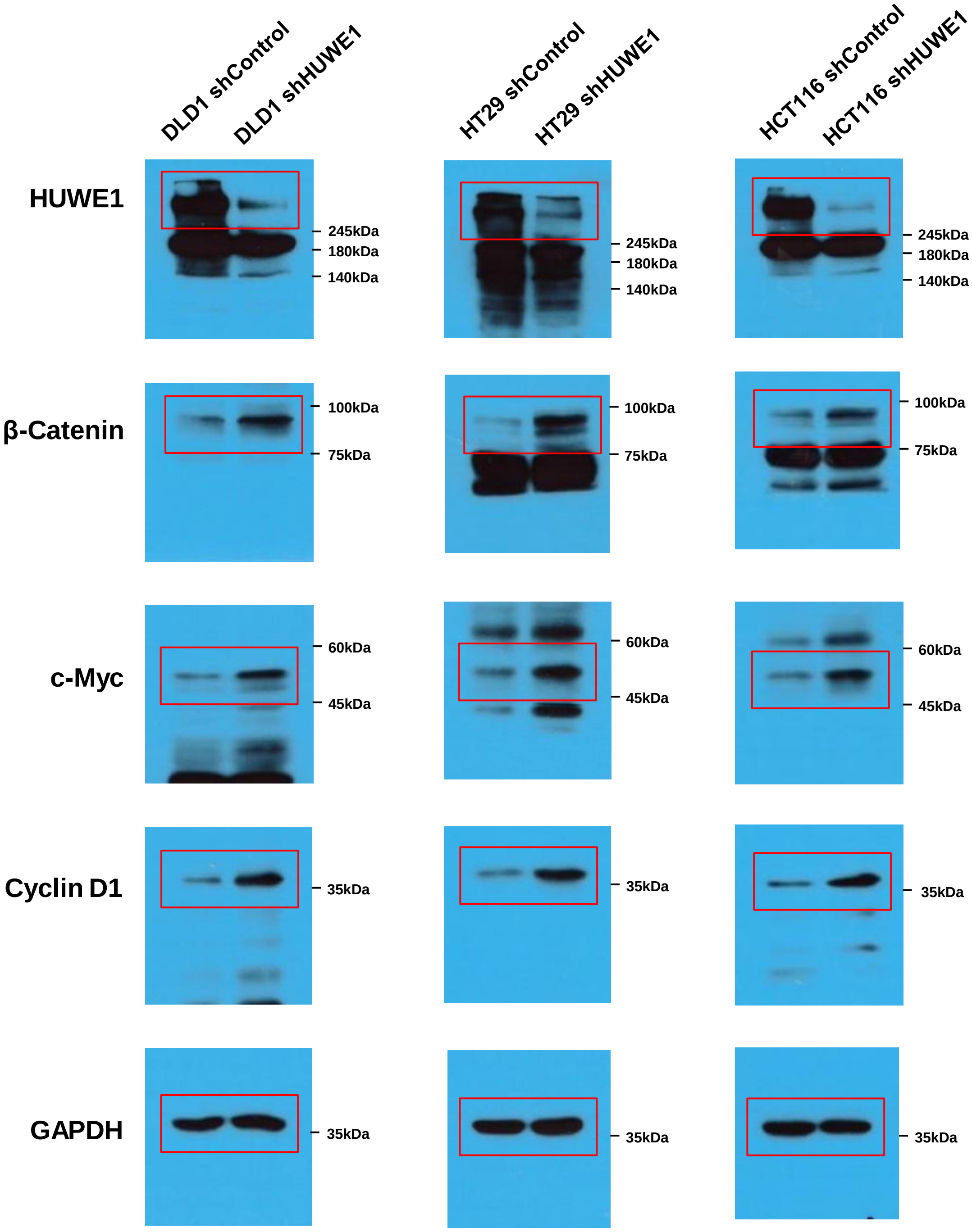

DLD1 shControl
DLD1 shHUWE1
HT29 shControl
HT29 shHUWE1
HCT116 shControl
HCT116 shHUWE1
HUWE1
β-Catenin
c-Myc
Cyclin D1
GAPDH
245kDa
180kDa
140kDa
100kDa
75kDa
60kDa
45kDa
35kDa

Figure 3C

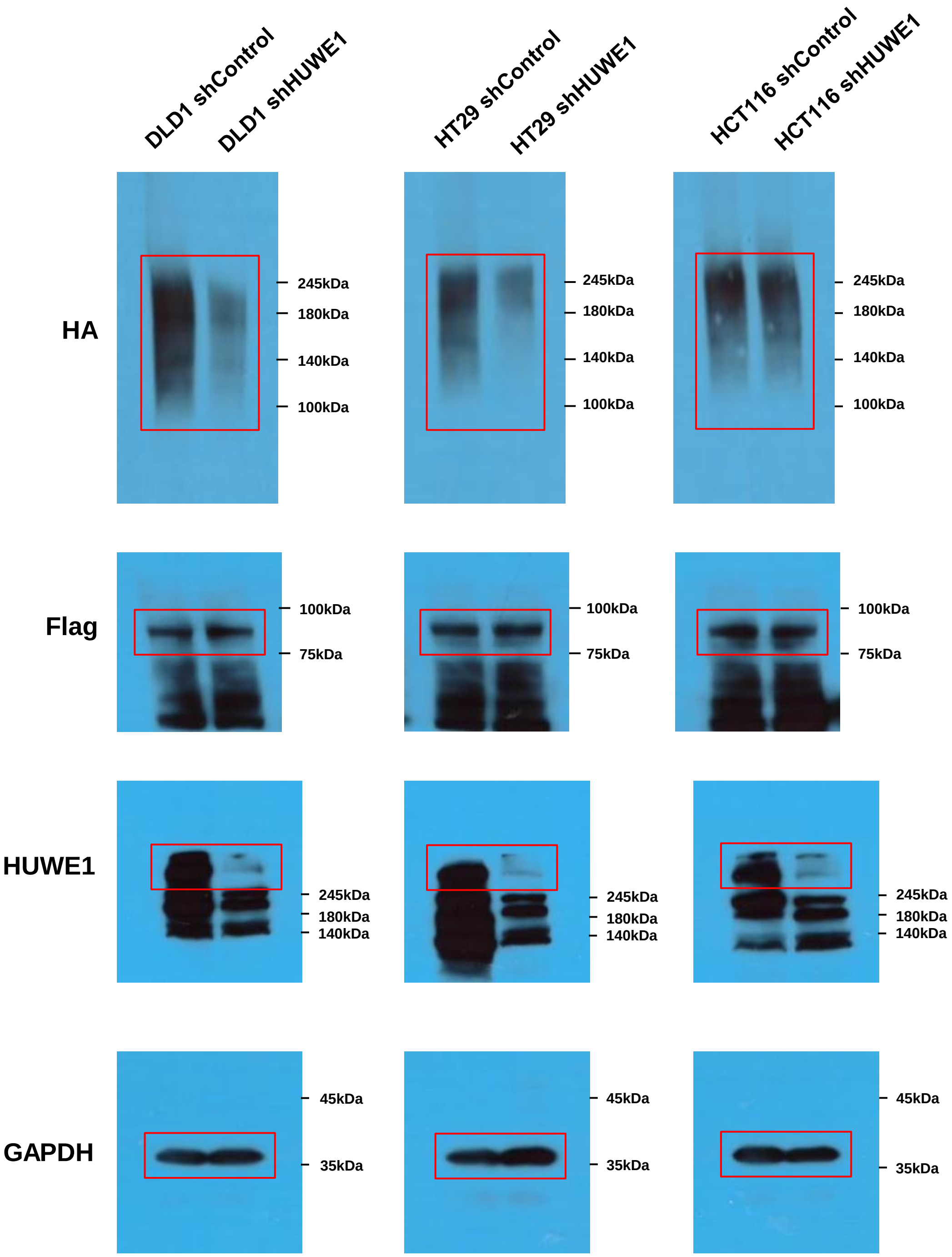
DLD1 shControl
DLD1 shHUWE1
HT29 shControl
HT29 shHUWE1
HCT116 shControl
HCT116 shHUWE1
HA
245kDa
180kDa
140kDa
100kDa
Flag
100kDa
75kDa
HUWE1
245kDa
180kDa
140kDa
GAPDH
45kDa
35kDa

Figure 3D

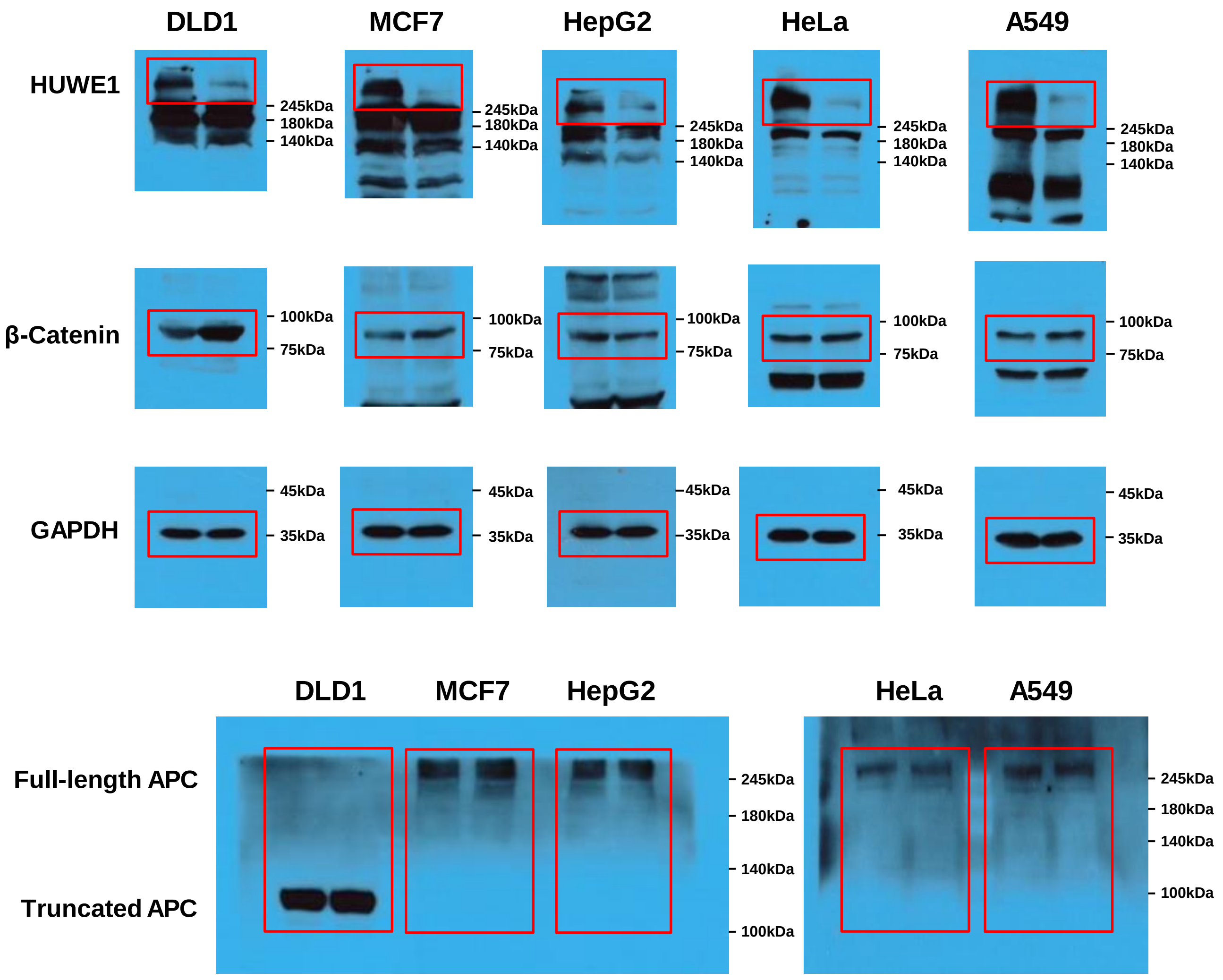
DLD1
MCF7
HepG2
HeLa
A549
HUWE1
β-Catenin
GAPDH
245kDa
180kDa
140kDa
100kDa
75kDa
45kDa
35kDa
Full-length APC
Truncated APC

Figure 3E

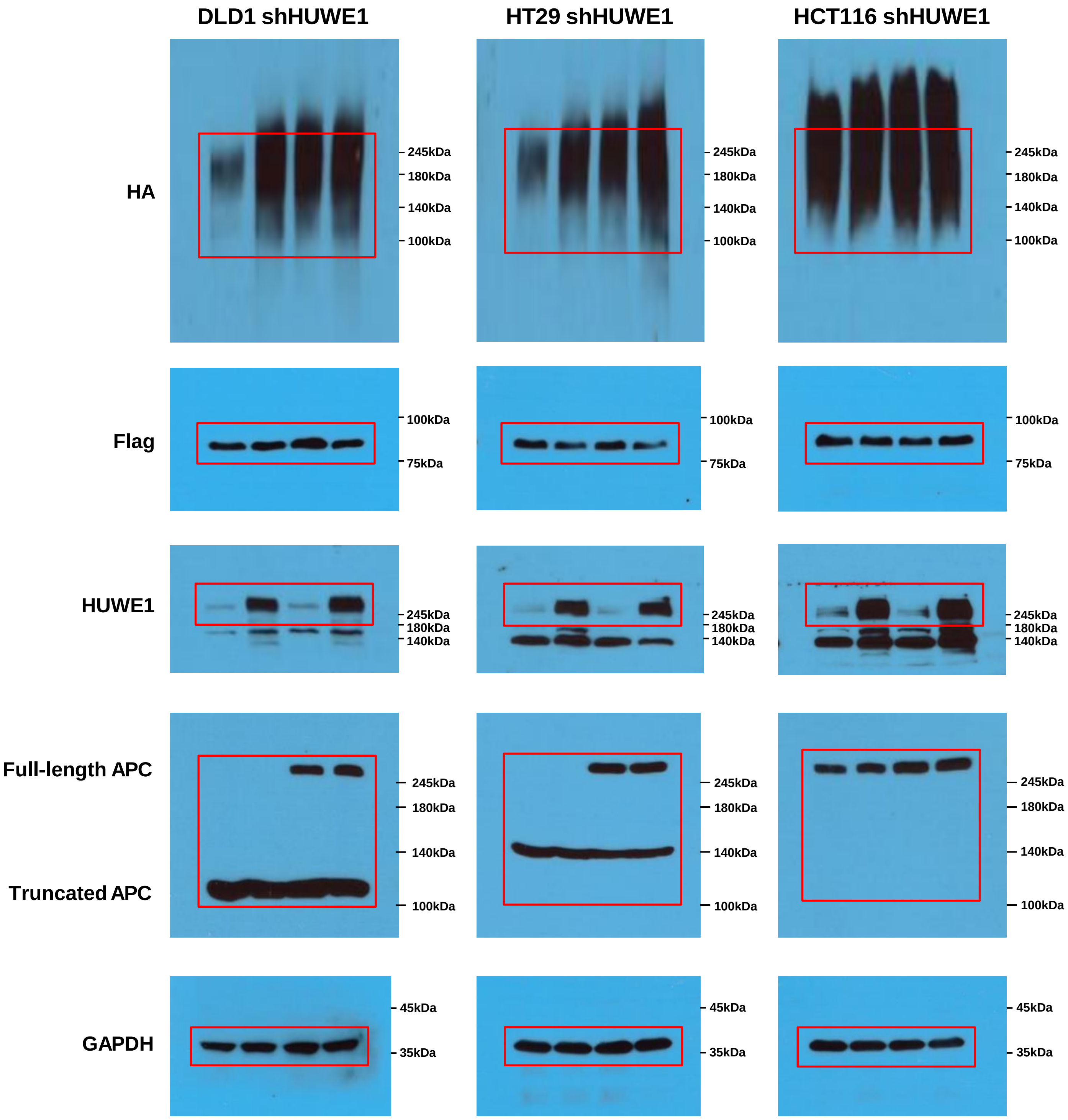
DLD1 shHUWE1
HT29 shHUWE1
HCT116 shHUWE1
HA
Flag
HUWE1
Full-length APC
Truncated APC
GAPDH
245kDa
180kDa
140kDa
100kDa
75kDa
45kDa
35kDa

Figure 3F

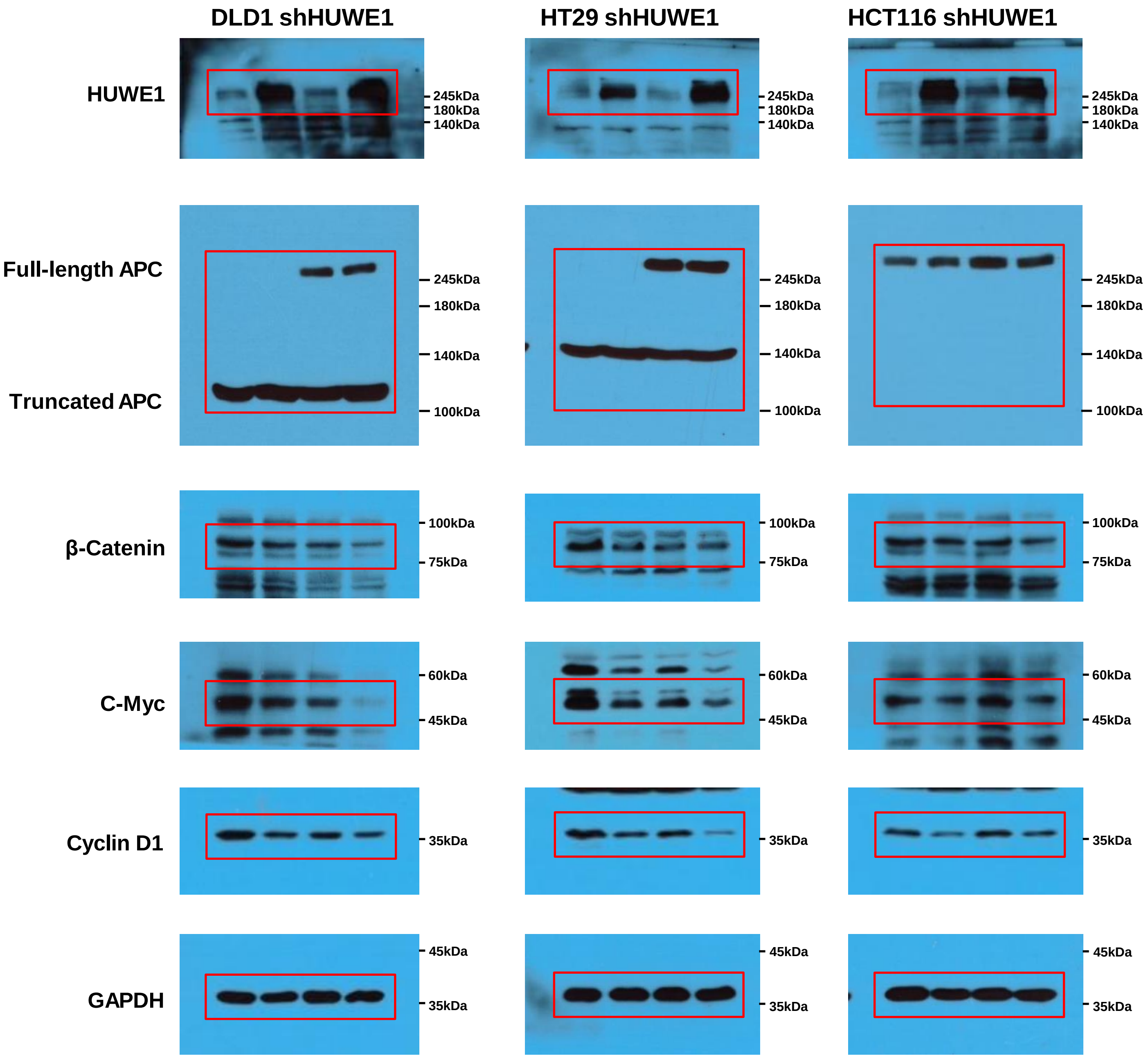
DLD1 shHUWE1
HT29 shHUWE1
HCT116 shHUWE1
HUWE1
245kDa
180kDa
140kDa
Full-length APC
Truncated APC
100kDa
β-Catenin
75kDa
C-Myc
60kDa
45kDa
Cyclin D1
35kDa
GAPDH

Figure 4A

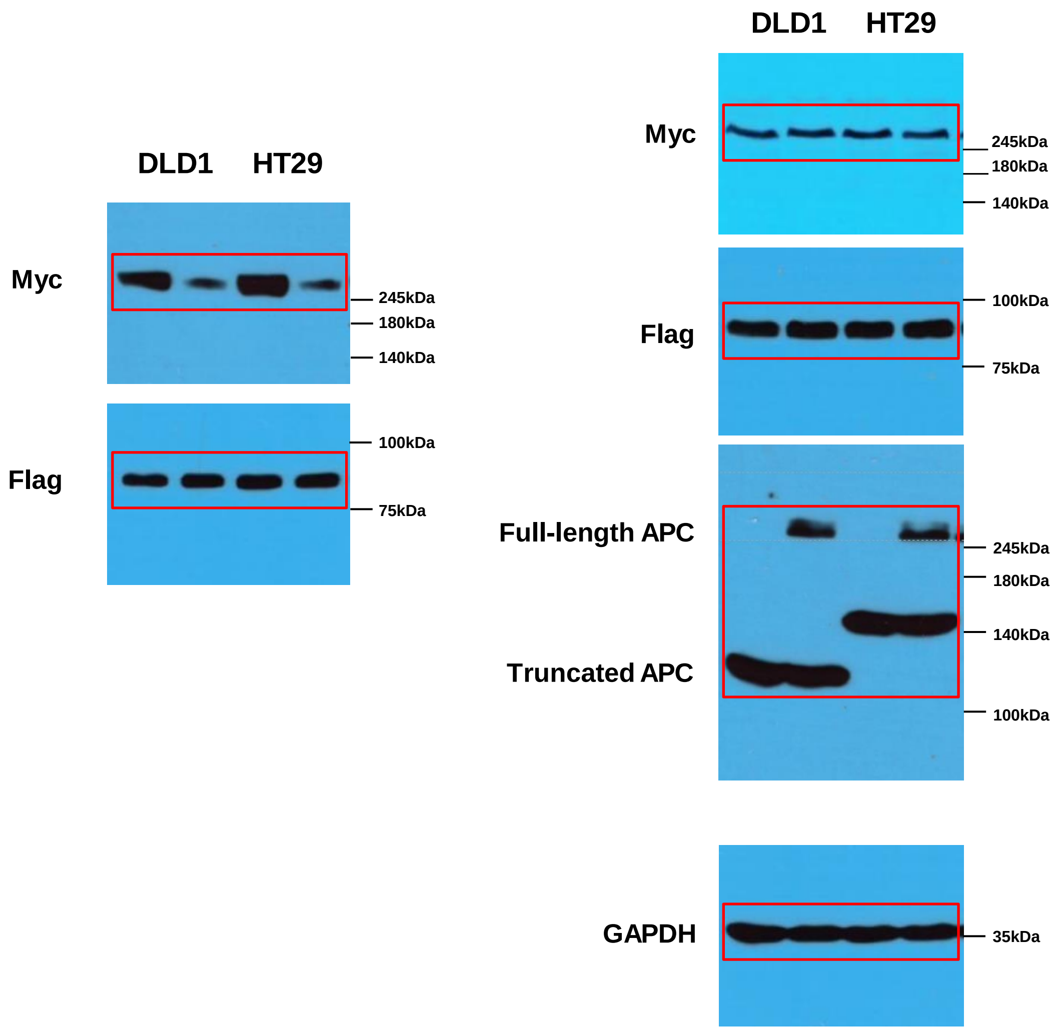
DLD1
HT29
Myc
245kDa
180kDa
140kDa
Flag
100kDa
75kDa
DLD1
HT29
Myc
245kDa
180kDa
140kDa
Flag
100kDa
75kDa
Full-length APC
Truncated APC
245kDa
180kDa
140kDa
100kDa
GAPDH
35kDa

Figure 4B

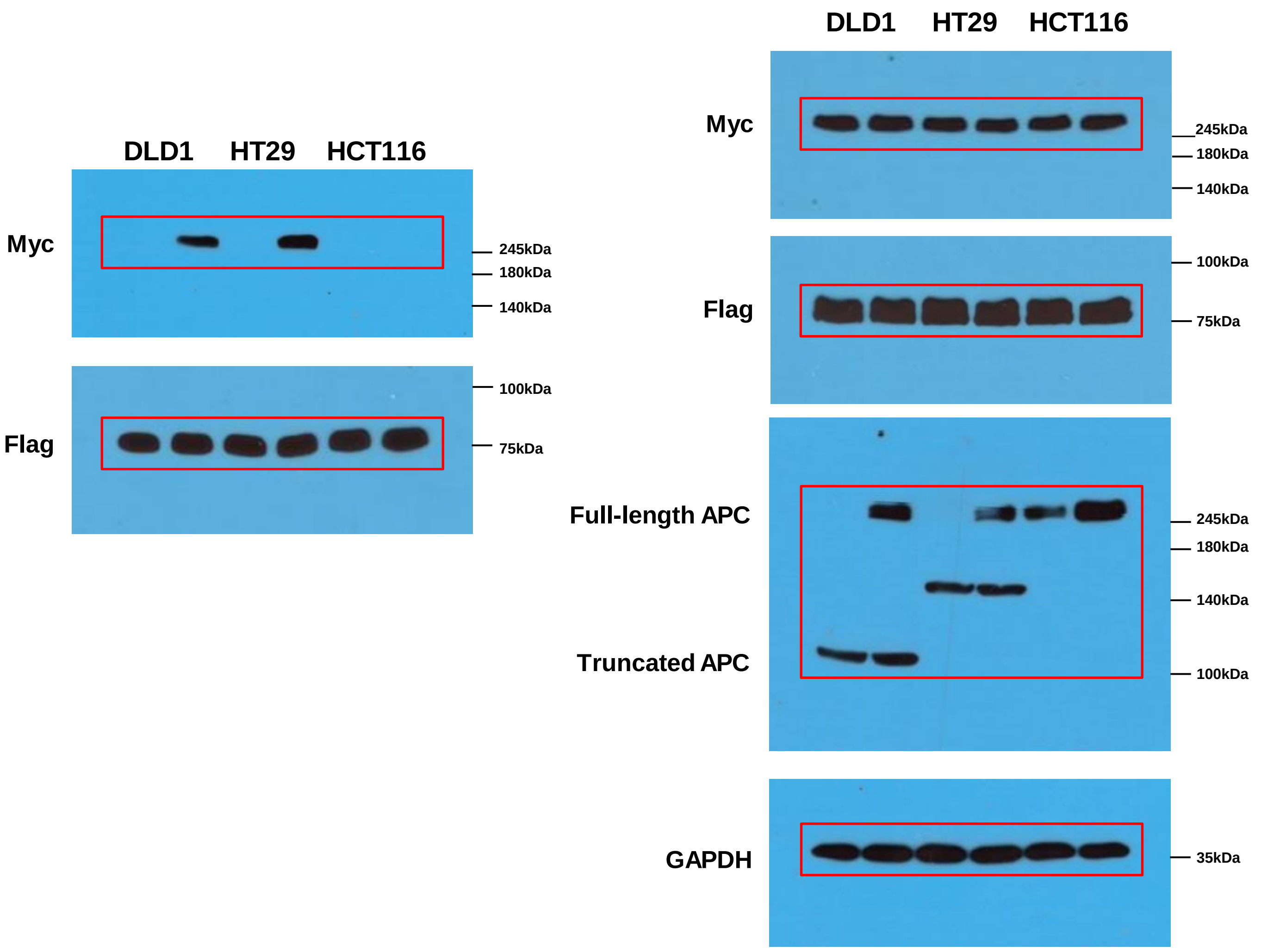
DLD1
HT29
HCT116
Myc
245kDa
180kDa
140kDa
Flag
100kDa
75kDa
DLD1
HT29
HCT116
Myc
245kDa
180kDa
140kDa
Flag
100kDa
75kDa
Full-length APC
Truncated APC
245kDa
180kDa
140kDa
100kDa
GAPDH
35kDa

Figure 4C

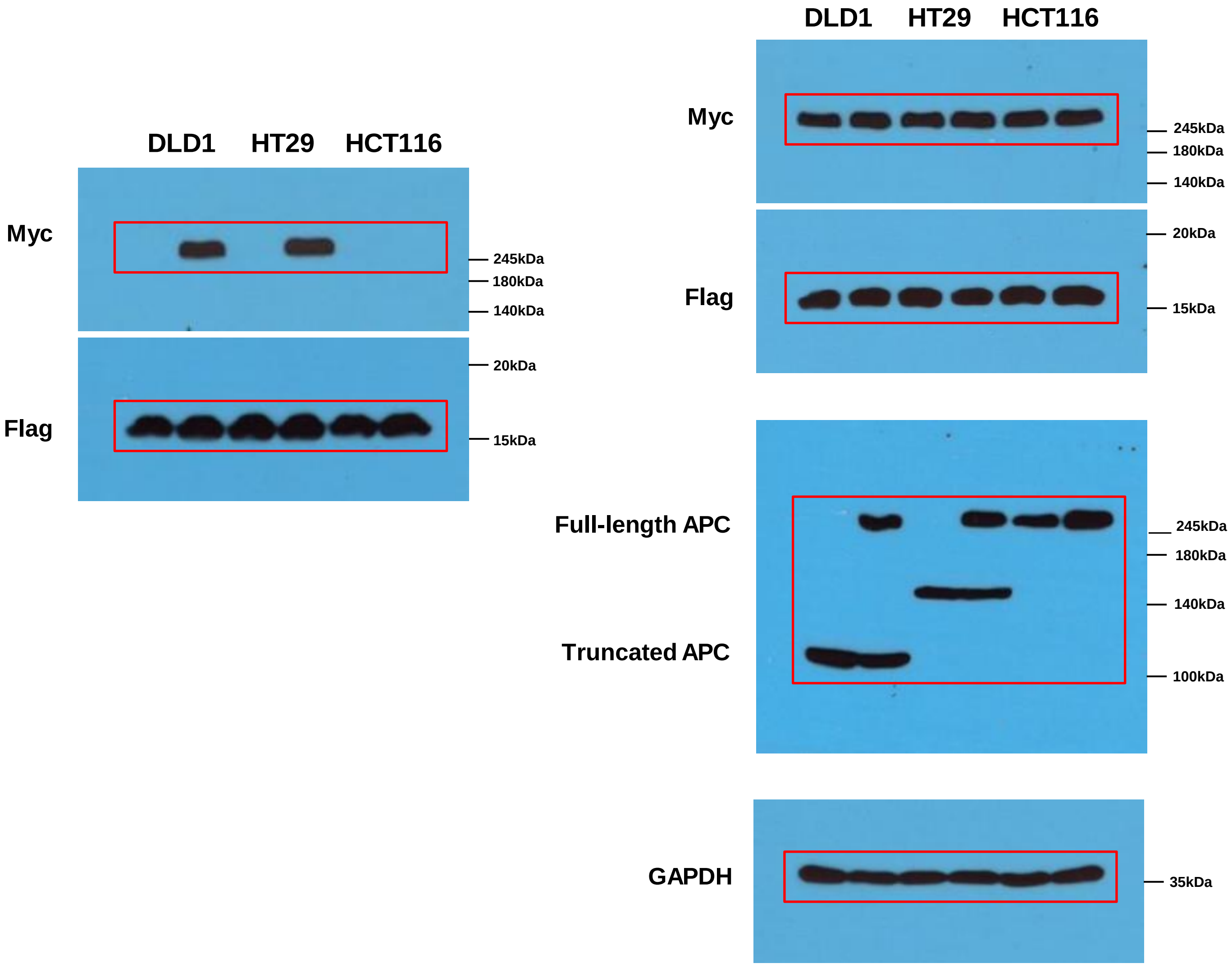

DLD1
HT29
HCT116
Myc
Flag
245kDa
180kDa
140kDa
20kDa
15kDa
DLD1
HT29
HCT116
Myc
Flag
245kDa
180kDa
140kDa
20kDa
15kDa
Full-length APC
Truncated APC
245kDa
180kDa
140kDa
100kDa
GAPDH
35kDa

Figure 5G

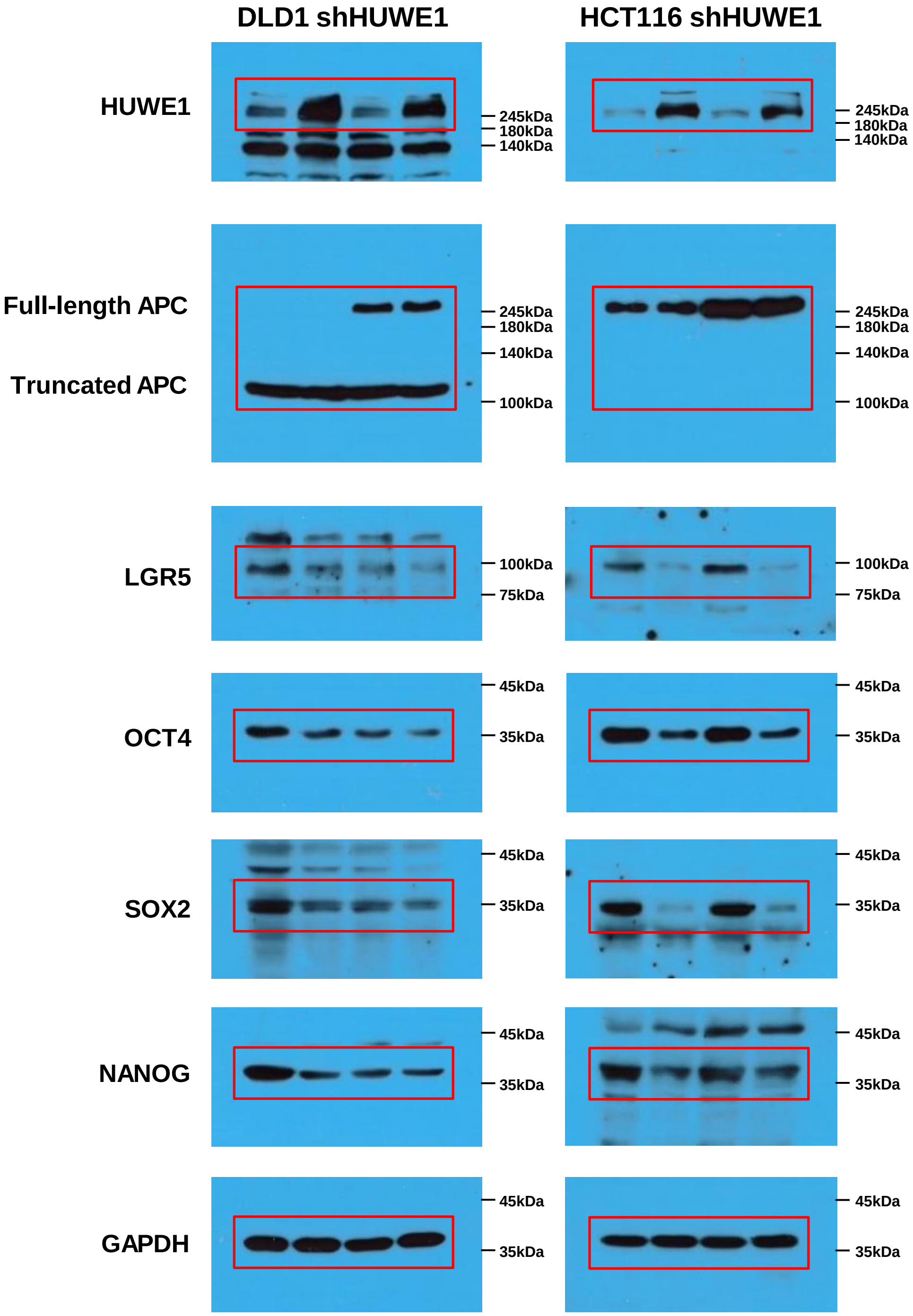

DLD1 shHUWE1
HCT116 shHUWE1
HUWE1
Full-length APC
Truncated APC
LGR5
OCT4
SOX2
NANOG
GAPDH
245kDa
180kDa
140kDa
100kDa
75kDa
45kDa
35kDa

Figure 5H

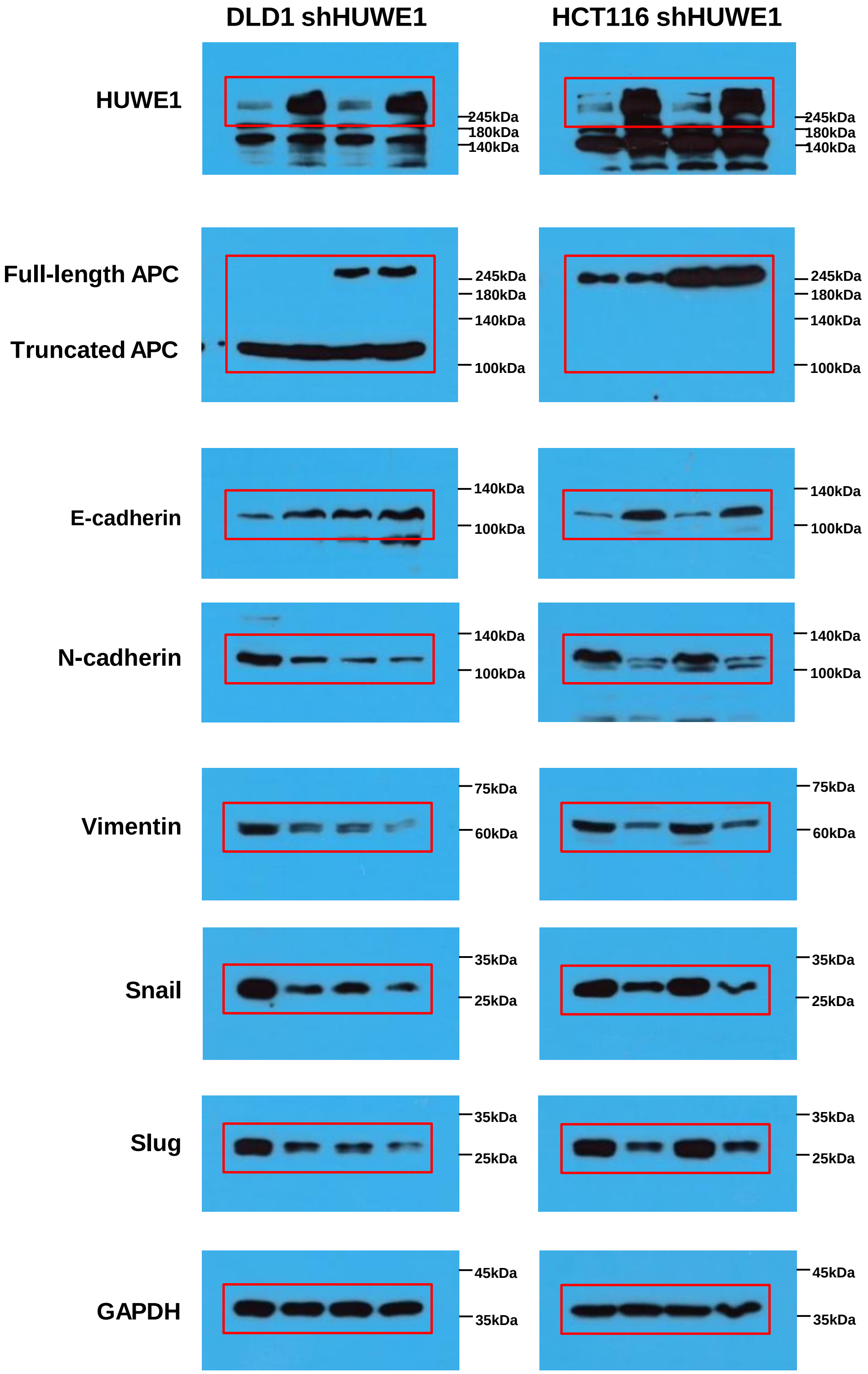
DLD1 shHUWE1
HCT116 shHUWE1
HUWE1
245kDa
180kDa
140kDa
Full-length APC
Truncated APC
245kDa
180kDa
140kDa
100kDa
E-cadherin
140kDa
100kDa
N-cadherin
140kDa
100kDa
Vimentin
75kDa
60kDa
Snail
35kDa
25kDa
Slug
35kDa
25kDa
GAPDH
45kDa
35kDa

Figure 6A

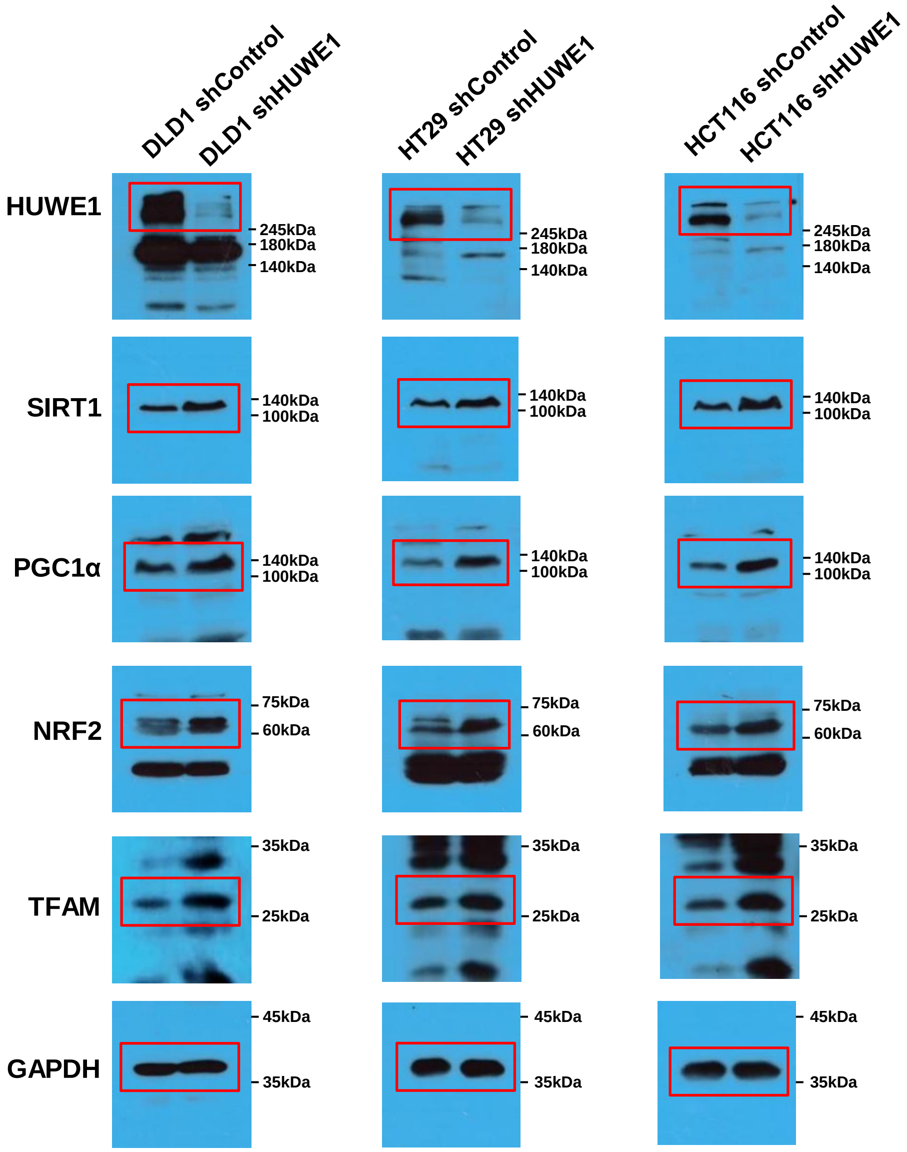

DLD1 shControl
DLD1 shHUWE1
HT29 shControl
HT29 shHUWE1
HCT116 shControl
HCT116 shHUWE1
HUWE1
245kDa
180kDa
140kDa
SIRT1
140kDa
100kDa
PGC1α
140kDa
100kDa
NRF2
75kDa
60kDa
TFAM
35kDa
25kDa
GAPDH
45kDa
35kDa

Figure 6B

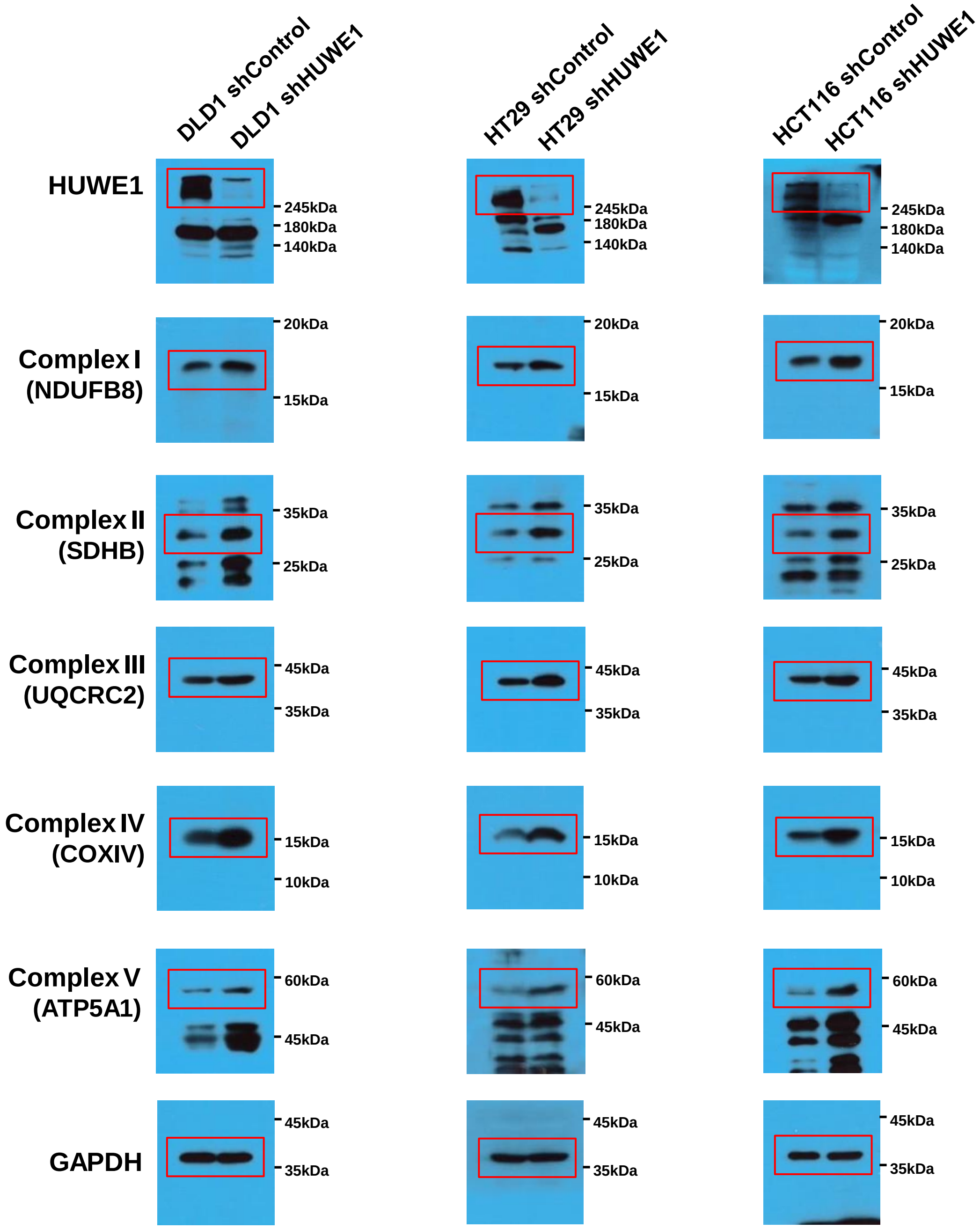

DLD1 shControl
DLD1 shHUWE1
HT29 shControl
HT29 shHUWE1
HCT116 shControl
HCT116 shHUWE1
HUWE1
245kDa
180kDa
140kDa
Complex I
(NDUFB8)
20kDa
15kDa
Complex II
(SDHB)
35kDa
25kDa
Complex III
(UQCRC2)
45kDa
35kDa
Complex IV
(COXIV)
15kDa
10kDa
Complex V
(ATP5A1)
60kDa
45kDa
GAPDH
45kDa
35kDa

Figure 6H

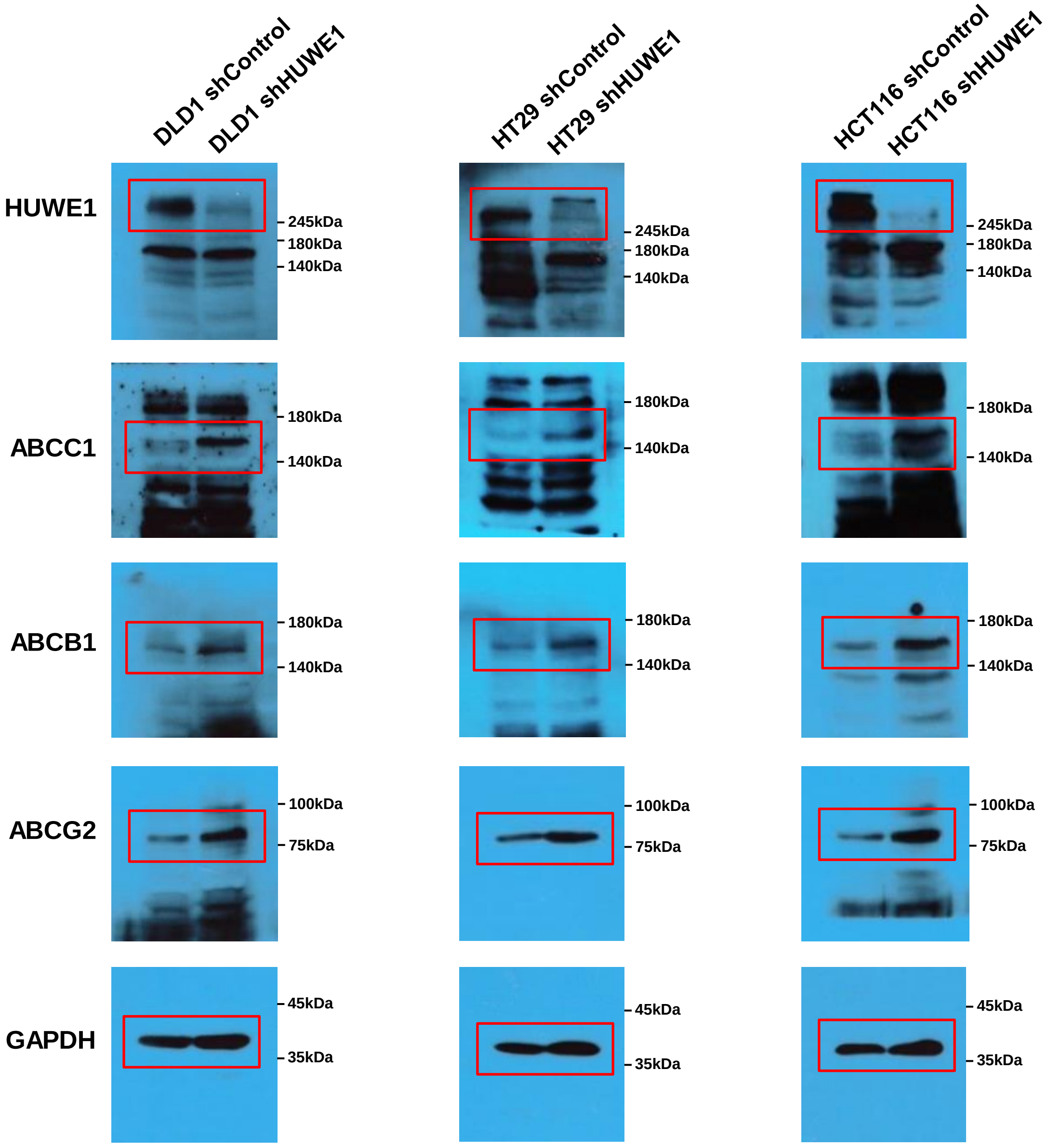
DLD1 shControl
DLD1 shHUWE1
HT29 shControl
HT29 shHUWE1
HCT116 shControl
HCT116 shHUWE1
HUWE1
ABCC1
ABCB1
ABCG2
GAPDH
245kDa
180kDa
140kDa
100kDa
75kDa
45kDa
35kDa

Figure 7A

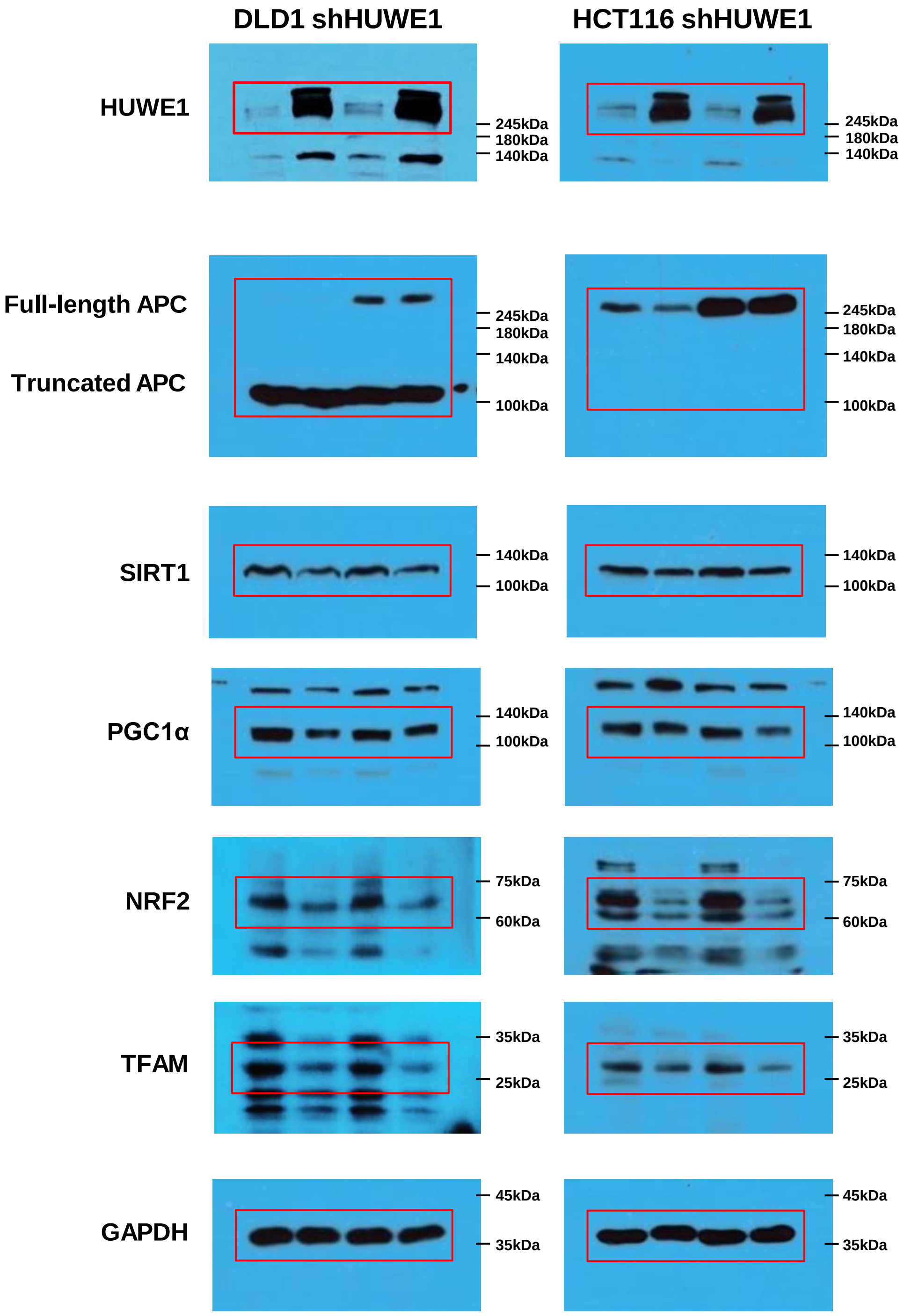

DLD1 shHUWE1
HCT116 shHUWE1
HUWE1
245kDa
180kDa
140kDa
Full-length APC
Truncated APC
245kDa
180kDa
140kDa
100kDa
SIRT1
140kDa
100kDa
PGC1α
140kDa
100kDa
NRF2
75kDa
60kDa
TFAM
35kDa
25kDa
GAPDH
45kDa
35kDa

Figure 7B

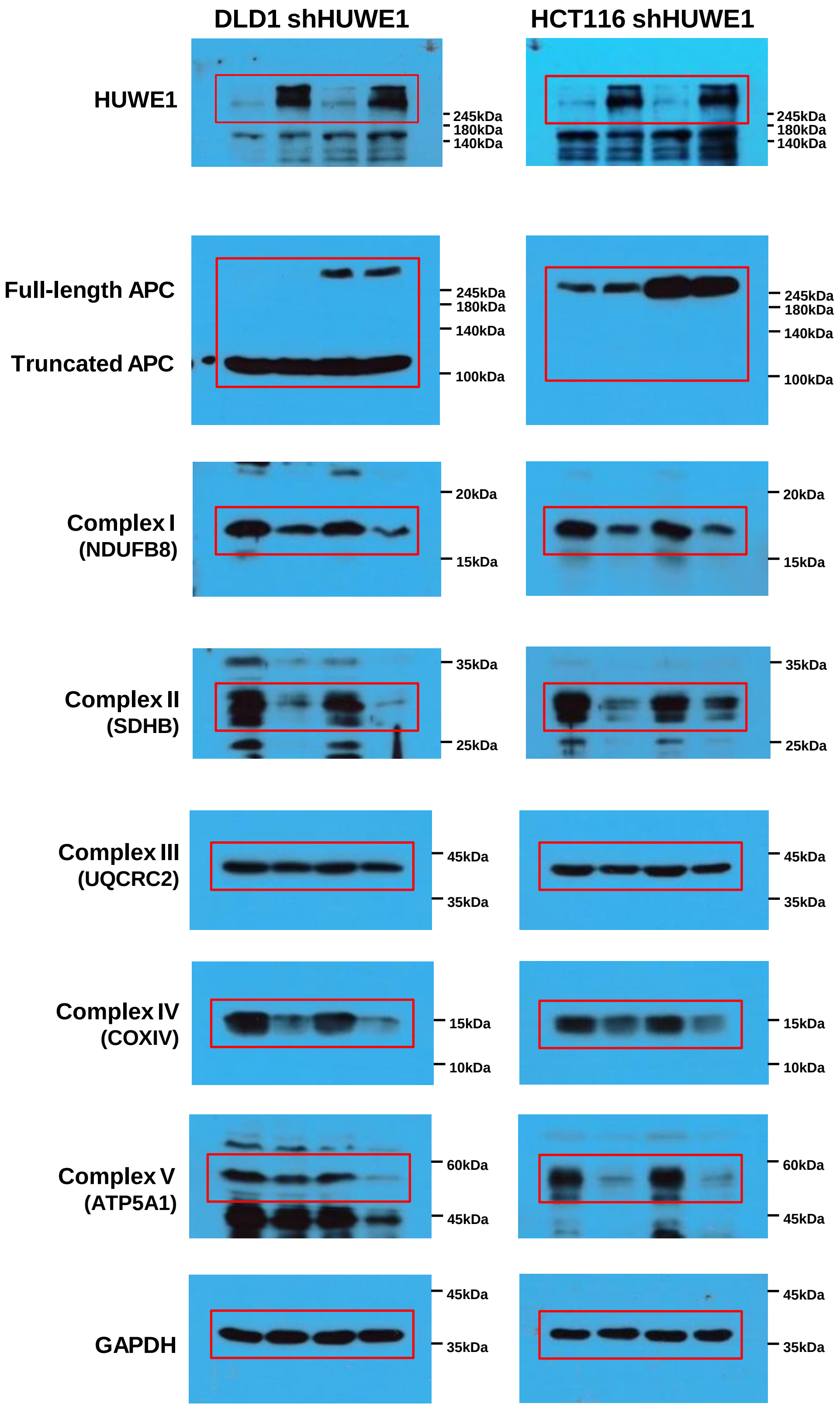

DLD1 shHUWE1
HCT116 shHUWE1
HUWE1
245kDa
180kDa
140kDa
Full-length APC
Truncated APC
100kDa
Complex I
(NDUFB8)
20kDa
15kDa
Complex II
(SDHB)
35kDa
25kDa
Complex III
(UQCRC2)
45kDa
Complex IV
(COXIV)
10kDa
Complex V
(ATP5A1)
60kDa
GAPDH

Figure 8B

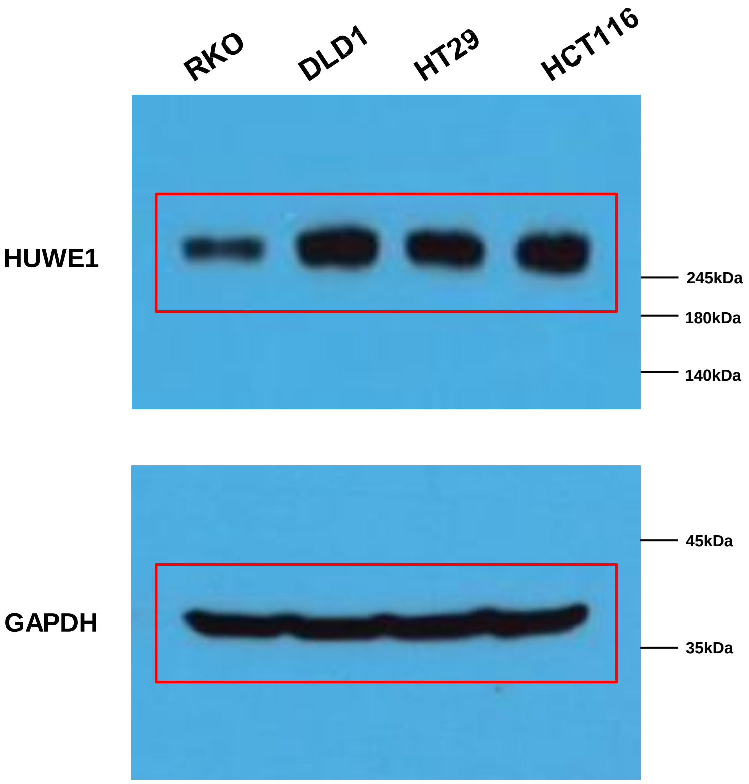
RKO
DLD1
HT29
HCT116
HUWE1
245kDa
180kDa
140kDa
GAPDH
45kDa
35kDa

Supplementary figure 3D

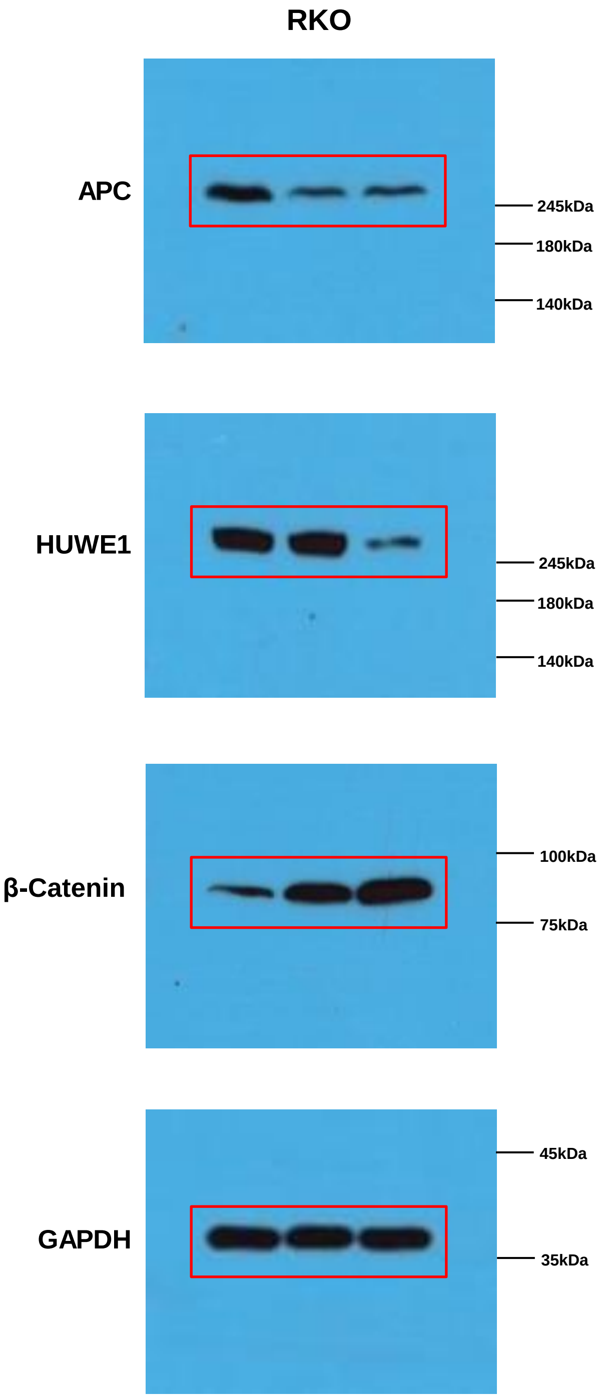

Supplementary figure 4

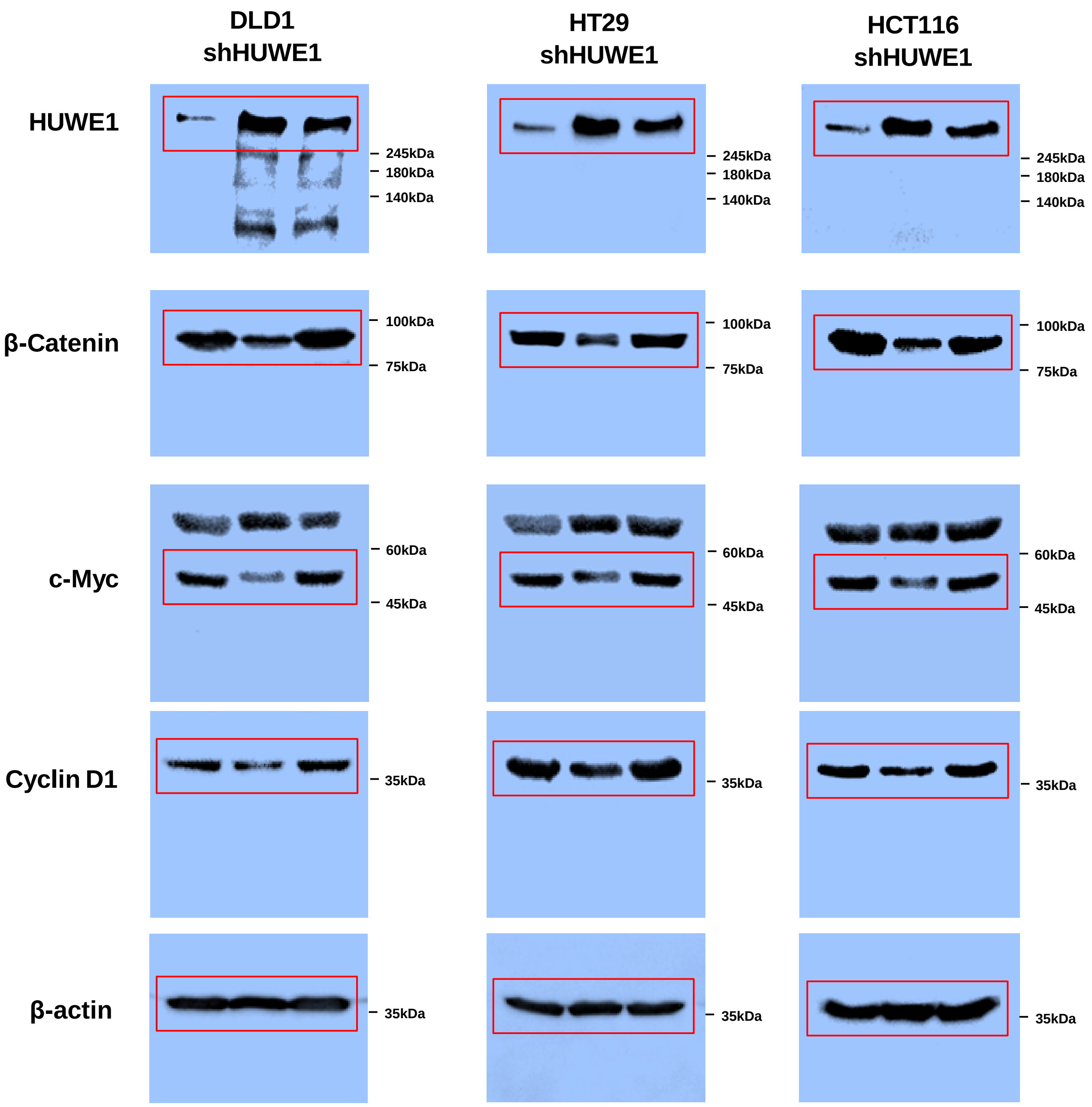

Supplementary figure 5A

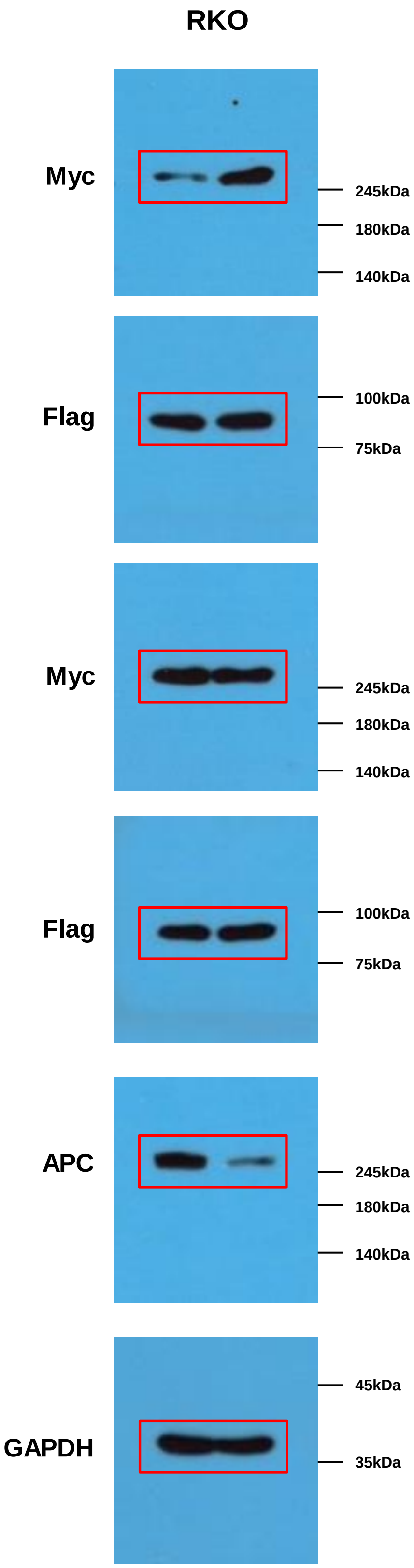

Supplementary figure 5B

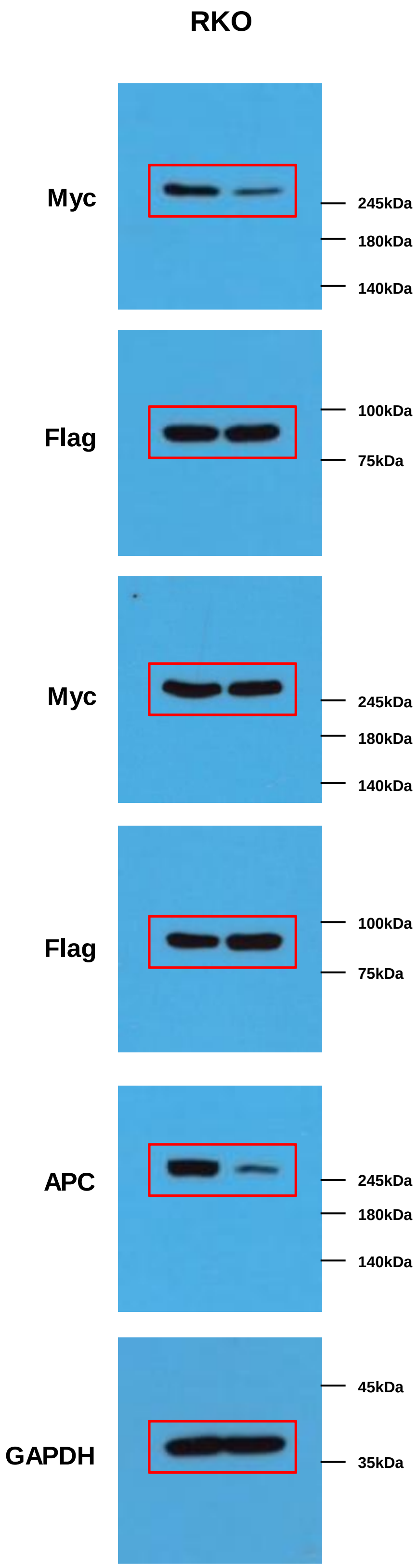

Supplementary figure 5C

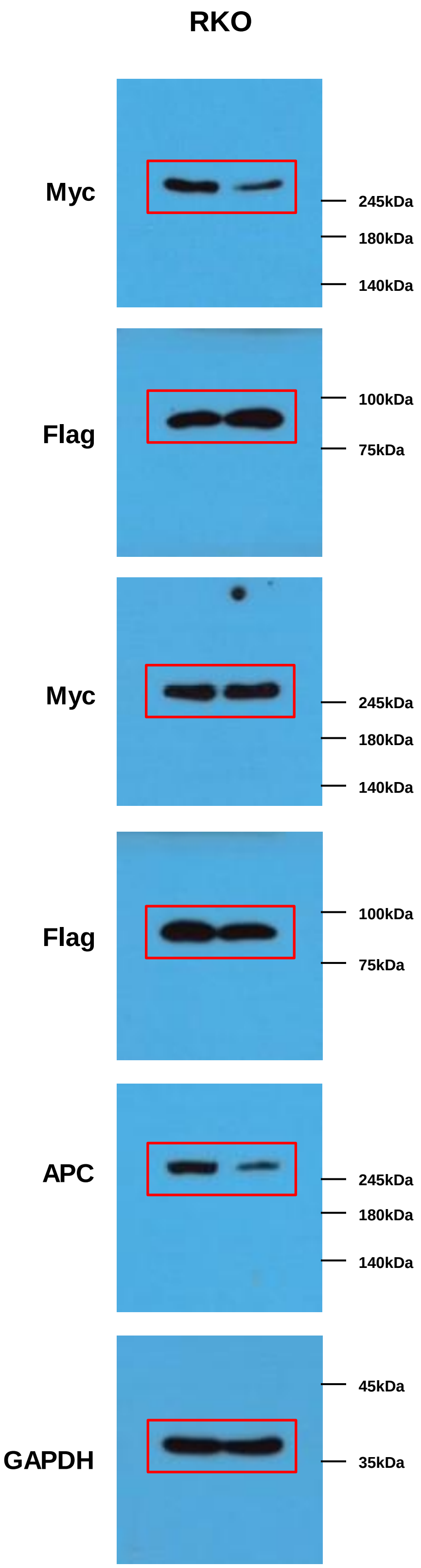

Supplementary figure 7

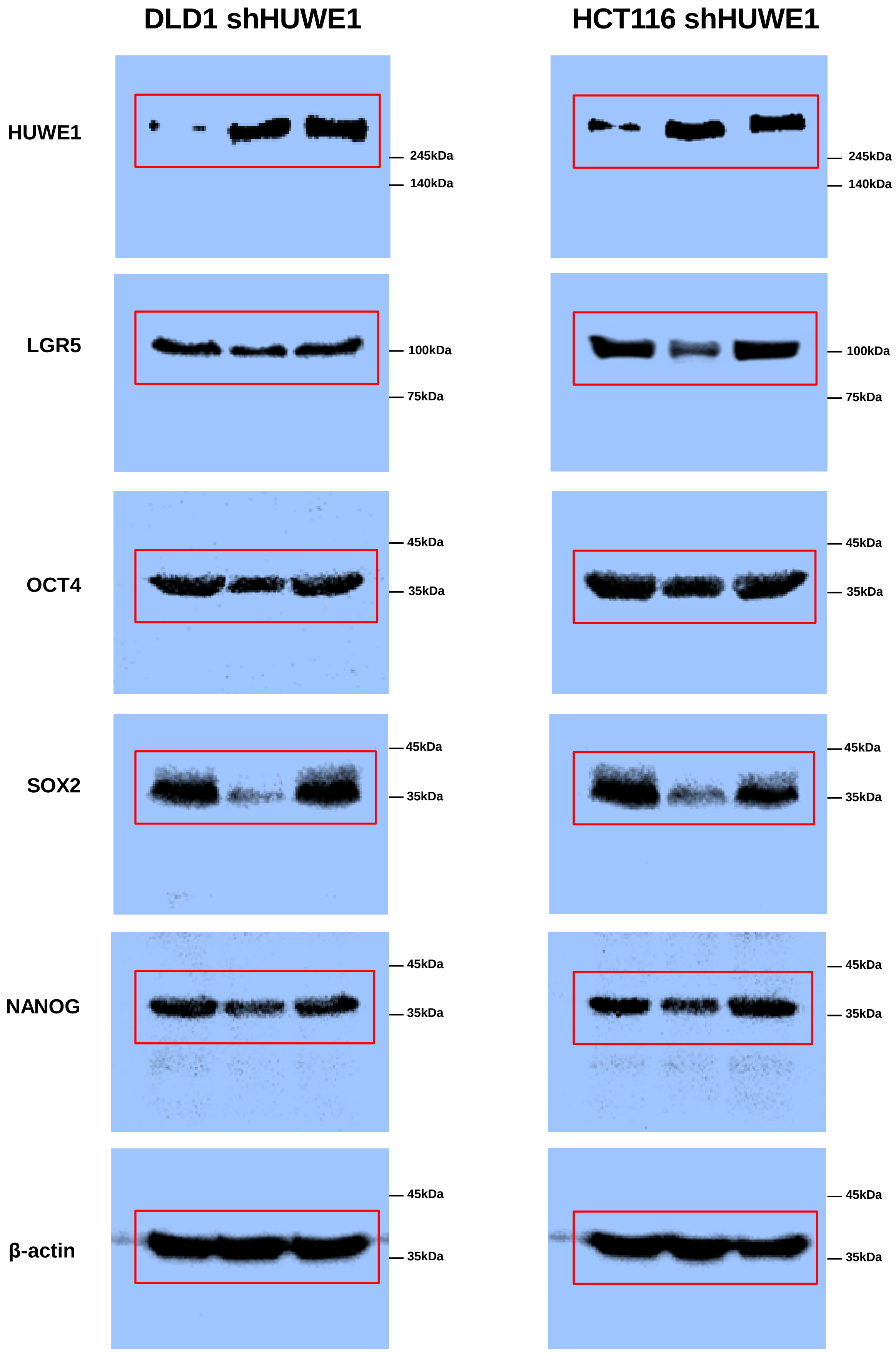

Supplement: Supplementary file 2 — Original Western blot [file 41420_2025_2731_MOESM2_ESM.pdf]
